# Efficient implicit constraint handling approaches for constrained optimization problems

**DOI:** 10.1038/s41598-024-54841-z

**Published:** 2024-02-27

**Authors:** Iman Rahimi, Amir H. Gandomi, Mohammad Reza Nikoo, Mohsen Mousavi, Fang Chen

**Affiliations:** 1 Data Science Institute, University of Technology Sydney, Sydney, NSW Australia; 2 University Research and Innovation Center (EKIK), Óbuda University, 1034 Budapest, Hungary; 3 Department of Civil and Architectural Engineering, Sultan Qaboos University, Muscat, Oman; 4 School of Civil and Environmental Engineering, University of New South Wales, Sydney, NSW Australia

**Keywords:** Constraint handling, Multi-objective optimization, Evolutionary algorithm, Boundary updating, Switching point, Engineering, Computational science

## Abstract

Many real-world optimization problems, particularly engineering ones, involve constraints that make finding a feasible solution challenging. Numerous researchers have investigated this challenge for constrained single- and multi-objective optimization problems. In particular, this work extends the boundary update (BU) method proposed by Gandomi and Deb (Comput. Methods Appl. Mech. Eng. 363:112917, 2020) for the constrained optimization problem. BU is an implicit constraint handling technique that aims to cut the infeasible search space over iterations to find the feasible region faster. In doing so, the search space is twisted, which can make the optimization problem more challenging. In response, two switching mechanisms are implemented that transform the landscape along with the variables to the original problem when the feasible region is found. To achieve this objective, two thresholds, representing distinct switching methods, are taken into account. In the first approach, the optimization process transitions to a state without utilizing the BU approach when constraint violations reach zero. In the second method, the optimization process shifts to a BU method-free optimization phase when there is no further change observed in the objective space. To validate, benchmarks and engineering problems are considered to be solved with well-known evolutionary single- and multi-objective optimization algorithms. Herein, the proposed method is benchmarked using with and without BU approaches over the whole search process. The results show that the proposed method can significantly boost the solutions in both convergence speed and finding better solutions for constrained optimization problems.

## Introduction

Constrained optimization problems arise naturally in most disciplines where finding optimized feasible solutions, especially with multiple objectives, is challenging. Despite the considerable variety of techniques developed in optimization fields and other disciplines to tackle these problems, the complexity of their solutions calls for alternative solution methods. Moreover, the computational cost has become another major concern due to the complexity of modern real-world problems. Also, the demand for optimal design and its applications in engineering and industry have become even more significant to satisfy the need for more strategic designs in modern engineering practices^1^. Engineering optimization problems usually consist of constraints that may be physical, geometrical, or operational; handling such constraints to find a single feasible solution is a challenging task^2,3^. For multi-objective problems, constraint handling is critical since a set of feasible solutions, i.e., Pareto front set, is sought. To tackle real-world constrained optimization problems, the constraint handling technique (CHT) has been combined with an evolutionary algorithm (EA) to achieve constrained evolutionary algorithm optimization (CEAO)^2^. Most CHTs proposed in the literature are explicit methods, e.g., CHTs by penalty or other fix-ups^2^.

The CHTs come in two main forms: explicit and implicit. Explicit techniques involve the explicit definition of constraints as part of the problem formulation. They are designed to respect and enforce constraints explicitly, guiding the search towards feasible regions of the solution space and addressing violations directly.

For example, several existing CHTs in the literature are based on feasibility and infeasibility regions^4–6^, priority assignment^7^, and tournament selection and a selection operator^7,8^. Some studies also provide surveys of CHTs^8–11^. In contrast, implicit techniques do not necessitate the explicit form of constraints. They handle constraints inherently during the search for the optimal solution, avoiding the need for additional objective function evaluations. Implicit CHT simplifies the problem formulation, but it may face challenges in handling complex or nonlinear constraints effectively.

For instance, Diwekar and Rubin^12^ proposed an implicit constraint handling technique for the ASPEN chemical process, which is based on a mixed integer mathematical programming approach consisting of master and subproblems. Since most of the relations in the above-mentioned problems are implicit^12^, proposed a method for partitioning the variables to significantly decrease computational time.

Raghavan et al.^13^ developed an implicit constraint handling technique for optimization based on the proper orthogonal decomposition (POD) of shapes, further producing a bi-level reparameterization approach for structural geometries. Uemura et al.^14^ proposed a real-coded genetic algorithm for implicitly constrained black-box optimization, in which the method employs the weighted mean of the best individuals in a population to find the optimal solution.

Mirabel and Lamiraux^15^ proposed a method to handle all constraints explicitly and implicitly that deals with manipulation planning, where the constraints are solved explicitly as much as possible. Implicit handling is alternatively employed with few variables in case all constraints cannot be handled explicitly. Nomura et al.^16^ introduced a natural evolution strategy called DX-NES-IC for implicit handling of constrained black-box optimization, which demonstrated better performance than DX-NES, xNES, and CMA-ES.

The current study extends the work of Gandomi and Deb^6^ by extending and applying boundary update (BU) to single-objective and multi-objective optimization problems (MOOPs). As novel strategies, two switching mechanisms are suggested that transform the landscape and variables to the original problem when the feasible region is found. Then, the optimization process is continued without the BU method.

In the work by Gandomi and Deb^6^, the authors proposed a novel approach, boundary update (BU), as an implicit CHT that updates variable bounds by directly using the constraints and then applying them to several single-objective optimization problems. Since, the BU method is an implicit CHT, it should be coupled with an explicit CHT, that is feasibility rules in our study^17^. As for the strategy without BU, only an explicit CHT (feasibility rules) is applied to solve the problem. The BU method is an implicit constraint handling approach that aims to cut the infeasible search space over iterations to find the feasible region faster. Although the BU method directs the search operators to the feasible space and reaches the first feasible solution reasonably fast, it twists the search space, making the optimization problem more challenging. The current study tries to tackle the above-mentioned problem by proposing two switching approaches.

The current study augments existing research by introducing two novel switching mechanisms. In the initial method, called Hybrid-cvtol, the BU approach is employed until constraint violations reach zero, ensuring zero violations across the entire population. Subsequently, the algorithm transitions to the optimization process without utilizing the BU approach. The second switching mechanism, called Hybrid-ftol, in this study involves employing the BU method during the initial phase until the objective space remains unchanged for a specified number of generations. At that point, the optimization problem shifts to a state without utilizing the BU approach.

The remainder of the paper is organized as follows. Section “Proposed approach” presents the proposed approach. Section “Research methodology” discusses the research methodology. Section “Numerical examples” provides numerical examples. Finally, Sects. “Discussion”, “Limitation”, and “Conclusion” give the discussion, limitation, and conclusion.

## Proposed approach

A constrained optimization problem can be formulated in Eqs. (1–3) as follows:

1 $$\mathrm{Maximize }({\text{Minimize}})\mathrm{ F}({\text{x}})=({{\varvec{f}}}_{1}\left({\varvec{x}}\right),\dots ,{{\varvec{f}}}_{{\varvec{t}}}\left({\varvec{x}}\right))$$

2 $${\text{s}}.{\text{t}}. {{\varvec{h}}}_{{\varvec{i}}}\left({\varvec{x}}\right)\le 0{\varvec{f}}{\varvec{o}}{\varvec{r}}{\varvec{i}}\in \{1,\dots ,{\varvec{n}}\}$$

3 $${{\varvec{g}}}_{{\varvec{j}}}\left({\varvec{x}}\right)=0\mathrm{for j}\in \{1,\dots ,{\varvec{m}}\}$$

4 $${\text{LB}}\le {\varvec{x}}\le {\varvec{U}}{\varvec{B}}$$

where $F(x)$ is the objective vector that consists of several objectives (*t* is the number of objective functions); *n* and *m* are the number of inequality and equality constraints, respectively; and *x* is the decision variable bounded by the lower bound (LB) and upper bound (UB) vectors. These equations yield several Pareto optimal solutions rather than producing a single solution^18^. In the case of single-objective optimization, a single solution can be found as a solution to $F\left(x\right)={f}_{1}\left(x\right)$. In the optimization process, the boundaries of decision variables, represented by lower bounds (LB) and upper bounds (UB) vectors, undergo dynamic changes as the algorithm progresses through iterations. This dynamic nature enables the optimization process to adapt and explore a continually evolving solution space, illustrating the inherent variability and adaptability of decision variable boundaries throughout the iterative stages. Hence, the iterative nature of optimization involves the dynamic evolution of the i-th decision variable boundaries throughout each iteration.

The proposed method uses the constraints to narrow down variable space and then forces the algorithm to focus its search in the feasible region by limiting the viable search space for the variable(s). In the BU method, the boundaries change iteratively and are updated during the optimization procedure. Mathematically, it could be written as follows^6^:

5 $$\exists i\in \left\{1,\dots ,m\right\}:[\forall j\in \left\{1,\dots ,n\right\}:{x}_{i}\ge {l}_{i,j}\left({x}_{\ne i}\right)\cup {x}_{i}\le {u}_{i,j}\left({x}_{\ne i}\right)]$$

where ${l}_{i,j}$ and ${u}_{i,j}$ are dynamic lower bound and upper bound for the *i*th decision variable, respectively. Updating the bounds involves the following scenarios:

6 $$\mathrm{If }{lb}_{i}=-\mathrm{\infty and }{ub}_{i}=+\infty :$$

7 $${lb}_{i}^{u}={l}_{i,j}({x}_{\ne i})$$

8 $${ub}_{i}^{u}={u}_{i,j}({x}_{\ne i})$$

Else:

9 $${lb}_{i}^{u}={\text{min}}({\text{max}}\left({l}_{i,j}\left({{\text{x}}}_{\ne {\text{i}}}\right),{lb}_{i}\right),{ub}_{i})$$

10 $${ub}_{i}^{u}={\text{max}}({\text{min}}\left({u}_{{\text{i}},{\text{j}}}\left({{\text{x}}}_{\ne {\text{i}}}\right),{ub}_{i}\right),{lb}_{i})$$

where ($${lb}_{i}^{u},{ub}_{i}^{u})$$ are updated boundaries. BU begins by selecting a repairing variable that can handle the most significant number of constraints without overlapping other repairing variables. As such, if there is more than one candidate, the one that handles the greatest number of constraints is selected. Also, if there is still another candidate for variable selection, one variable is chosen randomly^6^.

If a repairing variable handles the first ${k}_{i}$ constraints, then:

11 $${lb}_{i}^{u}={\text{min}}({\text{max}}[{l}_{i,1}\left({x}_{\ne i}\right),\dots ,{l}_{i,{k}_{i}}\left({x}_{\ne i}\right),{lb}_{i}],{ub}_{i})$$

12 $${ub}_{i}^{u}={\text{max}}({\text{min}}[{u}_{i,1}\left({x}_{\ne i}\right),\dots ,{u}_{i,{k}_{i}}\left({x}_{\ne i}\right),{ub}_{i}],{lb}_{i}) (\mathrm{where }{k}_{i}\le m)$$

If another repairing variable $({x}_{r})$ is defined:

13 $${lb}_{i}^{u}={\text{min}}({\text{max}}\left[{l}_{i,1}\left({x}_{\ne i,r}\right),\dots ,{l}_{i,{k}_{i}}\left({x}_{\ne i,r}\right),{lb}_{i}\right],{ub}_{i})$$

14 $${ub}_{i}^{u}={\text{max}}({\text{min}}\left[{u}_{i,1}\left({x}_{\ne i,r}\right),\dots ,{u}_{i,{k}_{i}}\left({x}_{\ne i,r}\right),{ub}_{i}\right],{lb}_{i})$$

15 $${lb}_{r}^{u}={\text{min}}\left({\text{max}}\left[{l}_{r,{k}_{i}+1}\left({x}_{\ne r}\right),\dots ,{l}_{r,{k}_{i}+{k}_{r}}\left({x}_{\ne r}\right),{lb}_{r}\right],{ub}_{r}\right)$$

16 $${ub}_{r}^{u}={\text{max}}\left({\text{min}}\left[{u}_{r,{k}_{i}+1}\left({x}_{\ne r}\right),\dots ,{u}_{r,{k}_{i}+{k}_{r}}\left({x}_{\ne r}\right),{ub}_{r}\right],{lb}_{r}\right)\mathrm{ where }r\in \left\{1,\dots ,n\right\} and r\ne i$$

In the BU method, a repairing variable could be substituted with a generalized semi-independent variable^6^ and then rewritten with lower and upper bounds as:

17 $$x\in \left\{{x}_{1},\dots ,{x}_{h},{x}_{h+1},\dots ,{x}_{n}\right\}\to mx\in \left\{{p}_{1},\dots ,{p}_{h},{x}_{h+1},\dots ,{x}_{n}\right\}$$

where ${p}_{1},\dots ,{p}_{h}$ are semi-independent variables; and *mx* indicates the set of selected repairing variables.

18 $$x\in \left\{{x}_{1},\dots ,{x}_{h},{x}_{h+1},\dots ,{x}_{n}\right\}\to mx\in \left\{{p}_{1},\dots ,{p}_{h},{x}_{h+1},\dots ,{x}_{n}\right\}$$

19 $${x}_{i}={lb}_{i}^{u}+{p}_{i}({ub}_{i}^{u}-{lb}_{i}^{u})\mathrm{ for i}=1, \dots ,\mathrm{ h where }0\le {p}_{i}\le 1$$

After selecting repairing variables, the search operator will be applied to the problem, the boundaries of non-repairing variables will be checked, and the $mx$-vector will be updated. During the solution procedure, the boundaries of repairing variables will be updated, and then the semi-independent variables (${p}_{i},i=1,\dots ,h)$ are remapped to the actual variables using updated boundaries. In the end, those constraints that were not involved in the repairing variable boundary, along with fitness values, will be evaluated using actual variables. Algorithm 1 illustrates the BU method in a constrained optimization problem.

**Algorithm 1:**
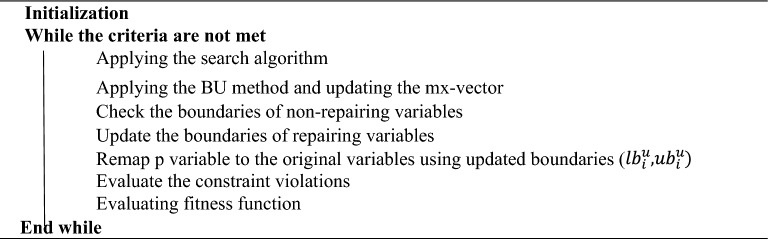
Implementation of the BU method in a constrained optimization problem.

As an illustrative example, the following multi-objective optimization problem (MOOP) is considered to explain BU:

20 $$\mathrm{min }{f}_{1}\left(x\right)=({x}_{1}^{2}+{x}_{2}^{2})$$

21 $$\mathrm{min }{f}_{2}\left(x\right)=-{\left({x}_{1}-1\right)}^{2}-{x}_{2}^{2}$$

22 $${g}_{1}\left({x}_{1}+{x}_{3}\right)={x}_{1}+{x}_{3}\le 1$$

23 $$-2\le {x}_{1}\le 2$$

24 $$-2\le {x}_{2}\le 2$$

25 $$-2\le {x}_{3}\le 2$$

Either ${x}_{1}\mathrm{ or }{x}_{3}$ can be selected as a repairing variable of the single constraint. All the variables are in the range of − 2 to 2. From the variable selection strategy^6^, as explained earlier, ${x}_{3}$ is selected as a repairing variable. The inequality constraint is solved with respect to ${x}_{3}$ as the lower and upper bound functions. The repairing variable (${x}_{3}$) is substituted with the mapped variable, $p$, in the range of 0–1. Therefore, the lower and upper bounds of the variables are {− 2, − 2, 0} and {2, 2, 1}, respectively. When the search algorithm is applied to the problem, the repairing variable is substituted with the mapped variable, and the boundary of the repairing variable is mapped for the rest of the search cycle^6^. The original variable can be calculated by the following formula to compute the objectives and constraints:

26 $${x}_{3}={lb}_{3}^{u}+{p}_{i}\times ({ub}_{3}^{u}-{lb}_{3}^{u})$$

where ${ub}_{3}^{u}\mathrm{ and }{lb}_{3}^{u}$ are the lower and upper bounds for the repairing variable ${x}_{3}$, respectively. The updated bounds can be written as follows:

27 $${lb}_{3}^{u}={\text{min}}\left(\mathit{max}\left({lb}_{3},1-{x}_{1}\right),{ub}_{3}\right)$$

28 $${ub}_{3}^{u}={\text{max}}\left(\mathit{min}\left({ub}_{3},1-{x}_{1}\right),{lb}_{3}\right)$$

**Figure 1:**
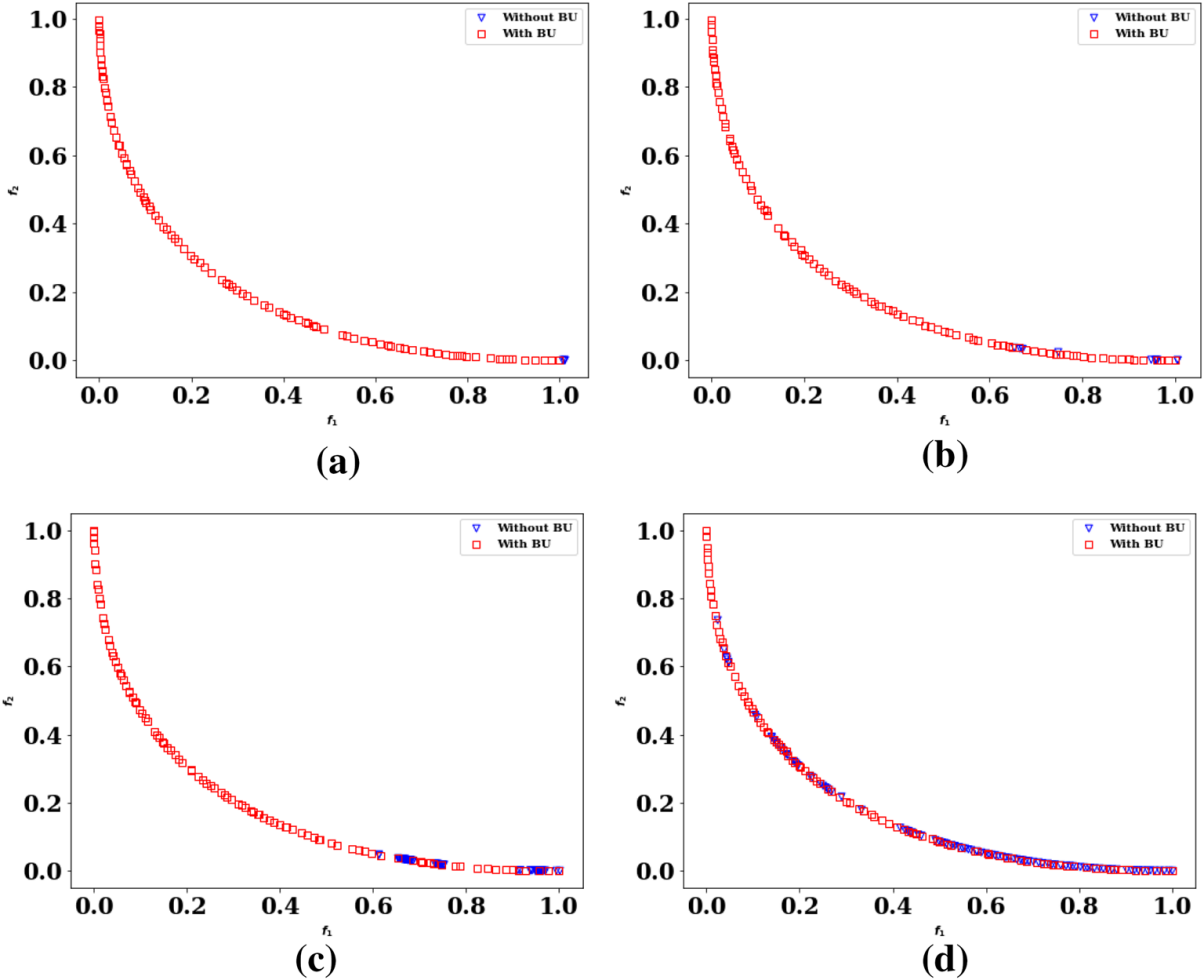
Pareto-optimal fronts obtained with and without the BU method.

The repairing variable is remapped to the actual boundary when the fitness values are evaluated. Figure 1a–d compare Pareto-optimal fronts found by the BU method coupled with a CHT (here, feasibility rules) and by the method without the BU method (only the feasibility rules approach is considered) for a population size of 100 for different generations. Figure 1 shows that the BU method produces more non-dominated solutions than the approach without BU. However, the number of non-dominated solutions is not always a good indicator for comparison. In the following sections, other indicators are explained in detail to compare the algorithms.

Although the BU method directs the search operator to the feasible space and reaches the feasible region in a reasonable time, it twists the search space, which can make the optimization problem more challenging and, hence, does not always bear good results and could potentially result in premature convergence. Consider the following example:

29 $$Max f\left(x\right)={x}_{1}^{2}+{x}_{2}^{2}-2\times {x}_{1}-2\times {x}_{2}+2$$

30 $$s.t. {g}_{1}\left(x\right):-\left(3\times {x}_{1}+{x}_{2}^{2}-5.5\right)\le 0$$

$${g}_{2}\left(x\right):-({x}_{1}^{2}+2\times {x}_{2}-4.5)\le 0$$

$${g}_{3}\left(x\right):0.8+{x}_{1}^{3}-{x}_{2}\le 0$$

31 $$0\le {x}_{1}\le 4$$

32 $$0\le {x}_{2}\le 4$$

**Figure 2:**
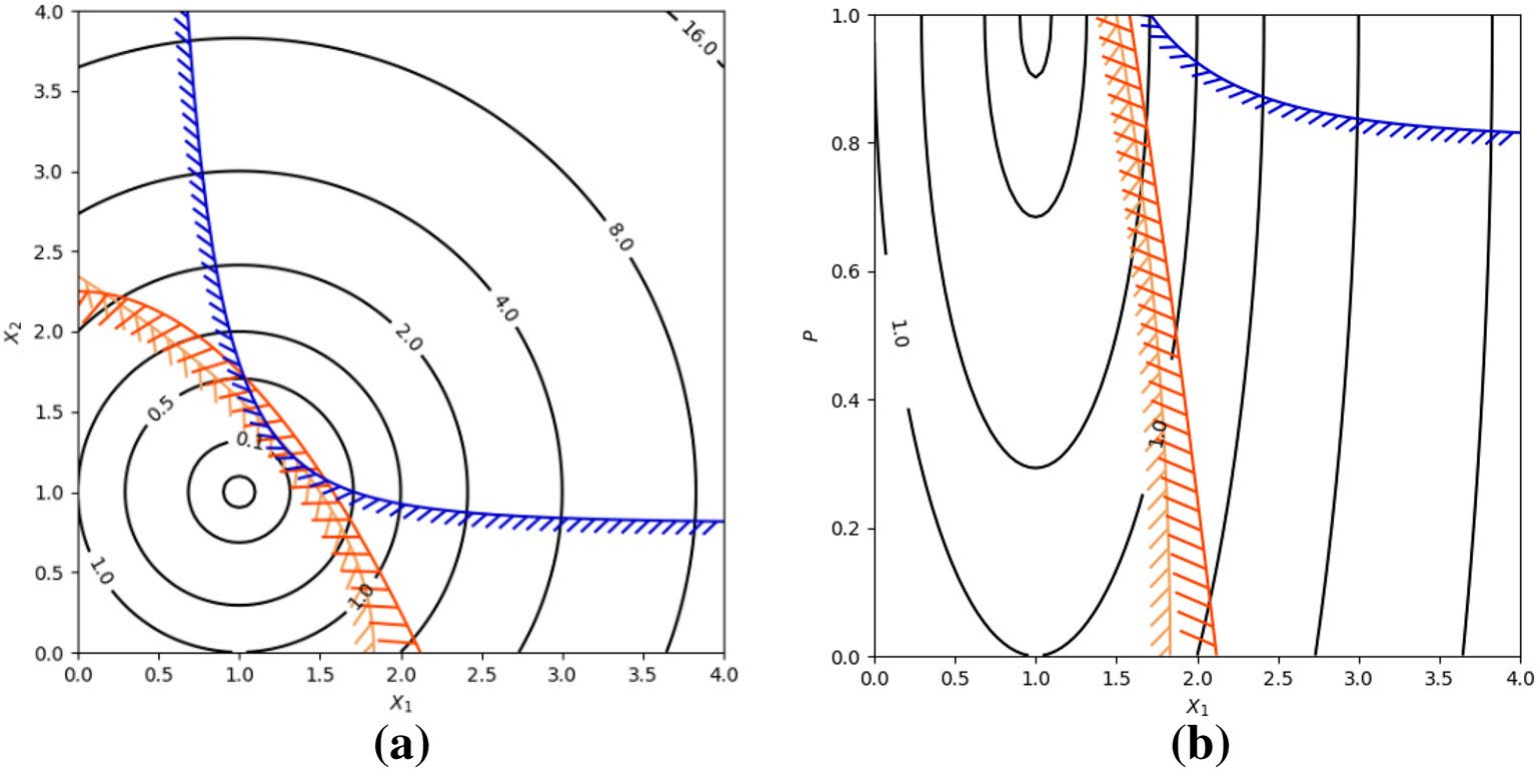
Contour plot of the search space.

In case of selecting variable ${x}_{2}$ as a repairing variable, the design space for the original variables ${x}_{1}$ and ${x}_{2}$ (Fig. 2a) is converted to the new design space (Fig. 2b). Twisting landscape may result in challenges for optimization problems, such as premature convergence. Some of these issues are presented in the following examples.

**Figure 3:**
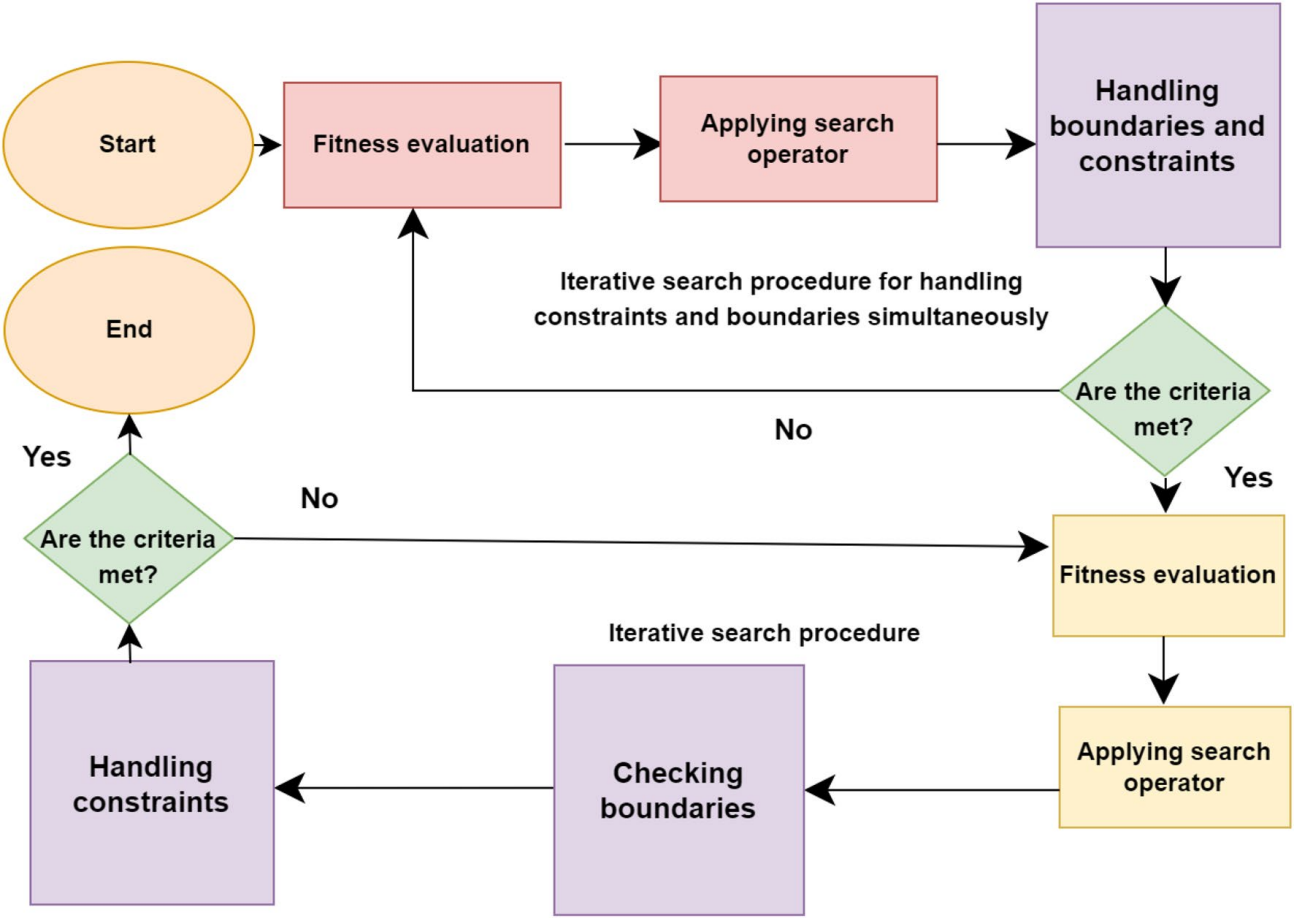
Optimization process of the proposed method.

Therefore, two distinct hybrid methods are introduced, each incorporating its own unique switching mechanism. In the initial approach, the BU method is employed until all constraint violations are reduced to zero, ensuring the absence of violations across the entire population. Subsequently, the algorithm transitions to the optimization process, excluding the use of the BU approach. In the second switching mechanism, the BU method is utilized in the initial phase until the objective space remains unchanged for a specified number of generations. Following this, the optimization problem transitions to a state where the BU approach is no longer employed. In both approaches, the optimization process is halted once the criteria and threshold tolerance are met. In the subsequent phase, the optimization problem is solved without the BU method, using the final population from the previous phase as the initial population for the new optimization problem. Figure 3 illustrates that the handling of boundaries and constraints occurs after the application of search operators.

During the first phase, the proposed methods simultaneously check constraints and examine variable boundaries, making the optimization process an iterative search procedure for handling constraints and boundaries concurrently. If the criteria are met, the second phase of the optimization process is conducted without utilizing the BU approach, constituting a sequential iterative search procedure where constraint handling occurs after boundary checking. The implementation of the proposed hybrid method is detailed in Algorithm 2.

**Algorithm 2:**
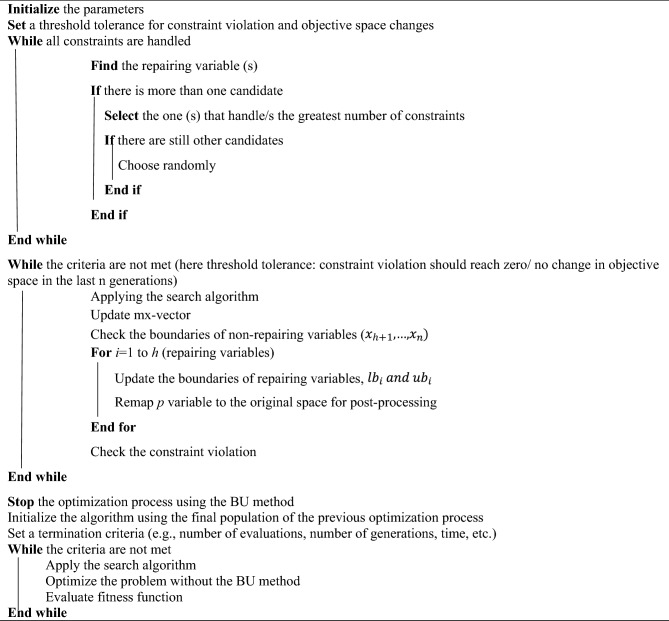
Implementation of the proposed method (hybrid methods).

## Research methodology

To evaluate the proposed method, Python and Pymoo libraries^19^ were used, which include well-stabilized algorithms. The specific algorithm parameters are presented in Table 1. Furthermore, because the implemented algorithms act stochastically, each of these algorithms were ran 31 times with random initial point(s).

**Table 1:** Specific parameter settings for the algorithms used in this study.

Algorithm	Parameter settings
Evolutionary strategy	Number of offspring = 200, rule = 1.0/7.0
Differential evolution	Variant = "DE/rand/1/bin", crossover constant (CR) = 0.3, Weighting factor (F) = 0.8
Stochastic ranking evolutionary strategy	Number of offspring = 200, rule = 1.0/7.0, gamma = 0.85, alpha = 0.2
Biased random key genetic algorithm	Number of elite = 200, number of offspring = 700, number of mutant = 100, bias = 0.7

Several examples, including benchmarks and real-world problems, were solved using three methods (without the BU method, with the BU method, and hybrid method). The presented examples are of different types: constrained by one, two, and three objective functions. For the problem with one objective function, the proposed method was implemented using evolutionary strategy (ES), differential evolution (DE), stochastic ranking evolutionary strategy (SRES)^20^, and biased random key genetic algorithm (BRKGA)^21^. For two objective functions, the BU method was applied using NSGA-II^17,22^. For three and five objective functions, some other EAs were used. As such, NSGA-III^23^, U-NSGA-III^24^, AGE-MOEA^25^, NSDE^26^, and NSDER^27^ were applied to optimization problems. The following metrics were evaluated to assess the accuracy of the obtained results.

- Number of non-dominated solutions: The BU method was coupled with an explicit constraint handling technique and compared to the approach without BU (only feasibility rules were applied to the optimization problem). Since the BU method aims to reduce the search space, the number of non-dominated solutions was determined for comparison.
- Constraint violation (CV): Compared to unconstrained optimization problems, constrained optimization problems are more challenging since a large proportion of infeasible regions appears in the search space (meaning the hit ratio is low), especially for highly constrained problems where these regions lead to some difficulties, such as feasibility-hardness and convergence-hardness. Therefore, even finding one feasible solution is a significant achievement. To test the effectiveness of the proposed method, the first feasible solutions found by the methods were compared and the whole population was tracked against generations.
- Performance indicators: Generational Distance (GD)^28^, Generational Distance Plus (GD+)^29^, Inverted Generational Distance (IGD)^30^, Inverted Generational Distance Plus (IGD+)^29^, and Hypervolume (HV) are the indicators that were analyzed.
- Running metric^31^: It is possible to trace the difference in the objective space followed by generations.

Moreover, the population was fixed at 100, and the maximum number of experiments was set to 11.

## Numerical examples

The proposed approach was applied to several single-objective and multi-objective optimization examples, including benchmarks and real-world problems. The presented examples include problems constrained by one, two, and three objective functions. The performance of the three strategies was evaluated using an example that has linear constraints. Following this numerical example, the proposed approach was evaluated to solve a surrogate model for a car-side impact design problem. This design problem consists of two versions of optimization, i.e., single- and multi-objective optimizations, and is an example of a black-box optimization problem. The speed reducer problem, a single-objective constrained engineering optimization problem, was also considered. For the multi-objective optimization problem, two benchmarks, namely OSY and BNH, and three real-world constrained optimization problems, including welded beam design, Cantilevered Beam design, and multi-objective car-side design problems were solved via the three strategies.

Several metaheuristic methods have been developed and implemented to reduce the total runtime of the optimization problems^2,32^, but still produce results that are almost as accurate and precise as conventional solving methods. Also, real-world engineering problems involve some type of optimization that is often constrained, most of which are considered MOOPs. No single solution exists for a MOOP; instead, different solutions generate trade-offs for various objectives. Furthermore, MOOPs arise naturally in most fields, and solving them has been a challenging problem for researchers^32,33^. EA methods have been identified as more effective in tackling the challenges that arise from MOOPs, for which the form of the Pareto-optimal front (discontinuity, nonconvexity, etc.) is not important^34,35^. Moreover, most multiobjective evolutionary algorithms (MOEAs) use the dominance concept^36,37^. In this work, for the single-objective optimization problems, Genetic Algorithm (GA), Differential Evolution (DE)^38^, Evolutionary Strategy (ES)^39^, Stochastic Ranking Evolutionary Strategy (SRES)^20^, and Biased Ranking Key Genetic Algorithm (BRKGA)^40^ were considered. NSGA-II^41^ is applied to bi-objective and multi-objective optimization problems with three objective functions and to multi-objective optimization problems with more than three objective functions, which are called many-objective optimization problems. NSGA-III^23^, U-NSGA-III^24^, and Adaptive Geometry Estimation-based Multi-objective Evolutionary Algorithm (AGE-MOEA)^25^ were further implemented for optimizing the problems. For all problems, the BU method was coupled with feasibility rules proposed by Deb^41^. Table 2 presents the studied examples in this work to assess the effectiveness of the proposed method in comparison with the other methods.

**Table 2:** Test problems considered in this study.

Problem	Type of problem	Example	No. of objective/s	No. of constraints	No. of variables
1	Benchmark	G1 problem^22^	1	9	13
2	Real-world	Single-objective car-side problem^42^	1	10	11
3	Real-world	Speed reducer^43^	1	11	7
4	Benchmark	BNH^5^	2	2	2
5	Benchmark	OSY^44^	2	6	6
6	Real-world problem	Welded beam problem^45^	2	4	4
**7**	Real-world problem	Cantilevered Beam design Problem^46^	2	10	10
8	Real-world problem	Multi-objective car-side problem^47^	3	10	7

### Single optimization problems

In this section, three single-optimization problems, namely G1, car-side design problem, and speed reducer, are considered to be solved using the different strategies discussed above.

#### G1 problem

**Figure 4:**
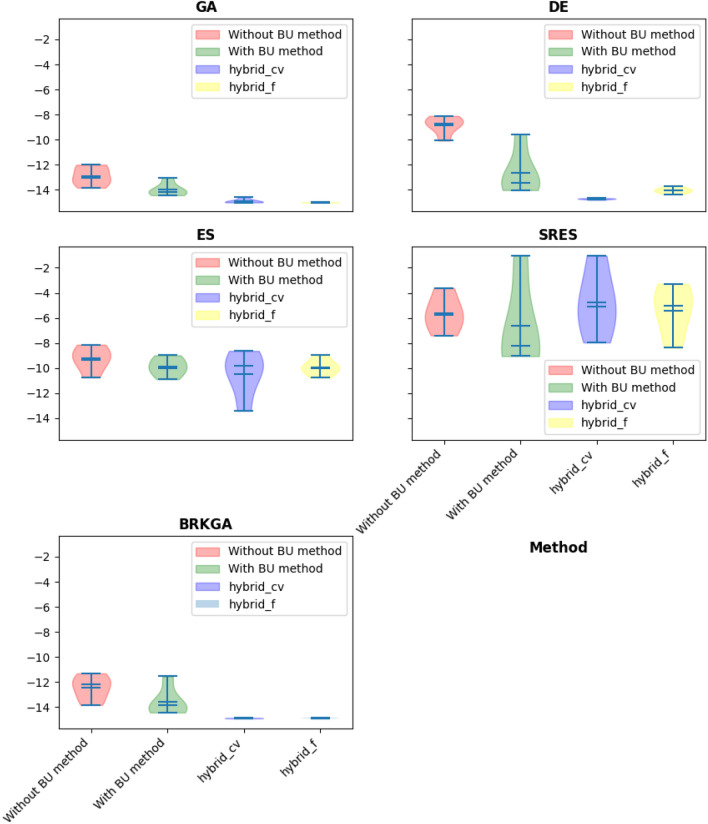
Violin plot of implementation of using different algorithms constraint handling methods (G01 problem).

The G1 problem is a famous mathematical constrained optimization problem that includes 13 decision variables, a quadratic objective function, and nine linear constraints. The problem is an excellent example of a highly linearly constrained problem, with a feasibility ratio of 0.111%^6^ (see the Appendix). In other words, it is a highly challenging to find a feasible solution for this constrained problem. The three different strategies were applied to this problem. According to the variable selection strategy, ${x}_{10}, {x}_{11}, and$ ${x}_{12}$ were selected as repairing variables for handling all nine constraints. The global optimum for this problem is -15, and the optimization results by using GA, DE, ES, SRES, and BRKGA with the BU method, without the BU method, and hybrid methods are presented in Tables 3, 4, 5 and 6 and Fig. 4. The Friedman rank test at a significant level of 5% was used to detect differences in treatments across test attempts (Table 4). Also, the Post-hoc Nemenyi Friedman test was performed to identify exactly which groups have different means (Tables 5, 6).

**Table 3:** Statistical results of different methods on G1 problem (The best performing method (objective function values) is marked in bold).

Algorithm	BU step	Best	Mean	Median	Worst	St. Dev
GA	None^a^	− 13.83	− 12.87	− 13.07	− 11.99	0.68
	BU	− 14.43	− 13.94	− 14.17	− 13.02	0.48
	Hybrid-cvtol	− **15.0**	− 14.87	− 14.95	− 14.58	0.15
	Hybrid-ftol	− **15.0**	− **14.99**	− **14.99**	− **14.99**	0.00
DE	None	− 10.08	− 8.83	− 8.74	− 8.10	0.66
	BU	− 14.02	− 12.61	− 13.46	− 9.55	1.62
	Hybrid-cvtol	− **14.80**	− **14.718**	− **14.710**	− **14.66**	0.047
	Hybrid-ftol	− 14.35	− 14.02	− 14.05	− 13.68	0.21
ES	None	− 10.75	− 9.24	− 9.38	− 8.13	0.89
	BU	− 10.89	− 9.89	− 9.99	− 8.98	0.65
	Hybrid-cvtol	− **13.41**	− **10.47**	− 9.84	− 8.63	1.63
	Hybrid-ftol	− 10.77	− 9.92	− **10.0**	− **8.98**	0.57
SRES	None	− 7.40	− 5.68	− 5.85	− **3.63**	1.24
	BU	− **9.05**	− **8.27**	− **8.18**	− 1.04	0.44
	Hybrid-cvtol	− 7.96	− 5.10	− 5.04	− 1.04	1.68
	Hybrid-ftol	− 8.38	− 5.51	− 4.54	− 3.27	1.84
BRKGA	None	− 13.83	− 12.46	− 12.20	− 11.30	0.89
	BU	− 14.43	− 13.56	− 13.85	− 11.54	1.03
	Hybrid-cvtol	− 14.90	− **14.88**	− **14.88**	− **14.86**	0.01
	Hybrid2-ftol	− **14.93**	− 14.87	− 14.88	− 14.85	0.02

^a^“None” means no BU method or hybrid is used and only feasibility rules as an explicit constraint handling method is worked alone.

**Table 4:** Summary of the p-value of the Friedman rank test over all runs.

Algorithm	p-value^a^	Statistic value
GA	0.002	14.62
DE	0.002	14.03
ES	0.06	7.28
SRES	0.25	4.07
BRKGA	0.005	12.59

^a^All statistical test in this study was performed at a significant level of 5%.

**Table 5:** Summary of the p-value of the posthoc_nemenyi_friedman over all runs.

Algorithm	Hybrid-cvtol vs. BU		Hybrid-cvtol vs. Without BU		BU vs. Without BU	
	p-value	h	p-value	h	p-value	h
GA	0.45	~	**0.03**	+	0.59	~
DE	0.12	~	**0.001**	+	0.45	~
ES	0.90	~	0.25	~	0.15	~
SRES	0.22	~	0.59	~	0.90	~
BRKGA	0.12	~	**0.01**	+	0.87	+

Posthoc_nemenyi_friedman: +, ~, and – presents the first method performs statistically significantly better, equal, and worse than the second approach.

Significant values are in bold.

**Table 6:** Summary of the p-value of the posthoc_nemenyi_friedman over all runs (continue).

Algorithm	Hybrid-ftol vs. BU		Hybrid-ftol vs. Without BU		Hybrid-ftol vs. Hybrid-cvtol	
	p-value	h	p-value	h	p-value	h
GA	0.12	~	**0.003**	~	0.87	~
DE	0.87	~	0.12	~	0.45	~
ES	0.90	~	0.06	~	0.90	~
SRES	0.59	~	0.90	~	0.90	~
BRKGA	0.20	~	**0.03**	+	0.90	~

Significant values are in bold.

The comprehensive analysis of algorithm performance on the G01 optimization problem, characterized by 13 continuous decision variables, nine linear constraints, and a quadratic objective function, unveils intricate relationships between problem features and algorithmic behavior. Each algorithm's response to the problem's unique attributes provides valuable insights into their adaptability and efficacy.

The hybrid methods, consistently showcasing superior performance across diverse algorithms, reflect an inherent versatility that aligns with the multi-faceted nature of the G01 problem. The distinct advantage of the hybridization approach becomes particularly evident in the handling of constraints. The BU method, serving as an implicit constraint-handling technique, demonstrates a keen ability to navigate the infeasible search space effectively. This characteristic proves invaluable in problems like G01, where constraints play a crucial role in shaping the feasible region. The hybrid methods, by strategically integrating the BU method, adeptly leverage this constraint-handling mechanism, enabling faster convergence to the feasible area.

**Figure 5:**
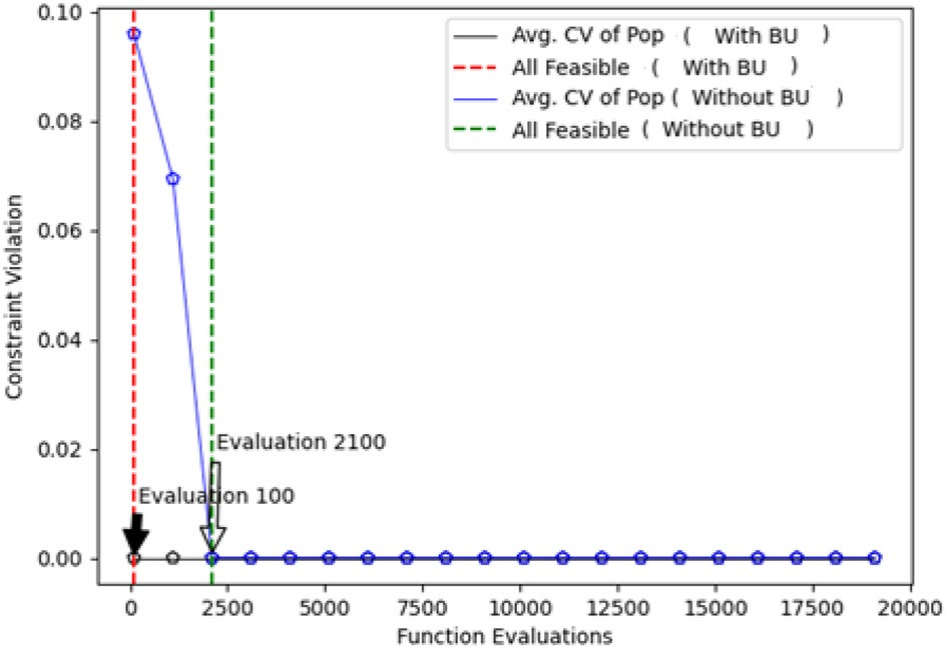
Constraint violation implemented by GA on the G01 test problem.

Considering the explicit constraints and the quadratic objective function in G01, it becomes apparent that the algorithmic adaptation to these features significantly impacts performance. Genetic algorithms (GA), when combined with the BU method (as hybrid methods), exhibit a noteworthy convergence pattern (as illustrated in Fig. 5). The initial phase, focused on constraints handling, ensures the algorithm converges to the feasible area efficiently. Subsequently, the transition to the optimization process without BU capitalizes on the algorithm's newfound knowledge of the feasible region, resulting in a streamlined path to the final solution. This adaptive approach to the problem's structure showcases how algorithmic behavior is intricately linked to specific features, allowing for a dynamic and context-aware optimization process.

The inadequacy of the SRES algorithm in this context sheds light on the sensitivity of algorithmic performance to the problem's characteristics. The SRES algorithm, designed for stochastic optimization, might face challenges when confronted with the structured nature of the G01 problem. The lack of significant improvement suggests that algorithmic suitability is not universal but rather contingent on the problem's intricacies. This observation underscores the importance of aligning algorithmic choices with the specific features and constraints of the optimization problem at hand.

Furthermore, the reduction in standard deviation observed in the hybrid methods implies a more stable and reliable convergence behavior. This heightened stability is particularly valuable in the presence of complex problem features, such as the quadratic objective function, where rapid fluctuations in convergence patterns might hinder progress. The hybrid methods' ability to maintain a consistent trajectory towards the optimal solution underscores their resilience in handling the intricacies introduced by specific problem characteristics.

In summary, the algorithmic performance on the G01 problem intricately relates to its unique features. The hybrid methods showcase adaptability and versatility in addressing constraints, demonstrating a keen understanding of the problem structure. The nuanced convergence patterns, strategic switching mechanisms, and sensitivity to problem-specific attributes provide valuable insights into the dynamic interplay between algorithmic behavior and the intricacies of the optimization problem.

#### Car-side problem

**Figure 6:**
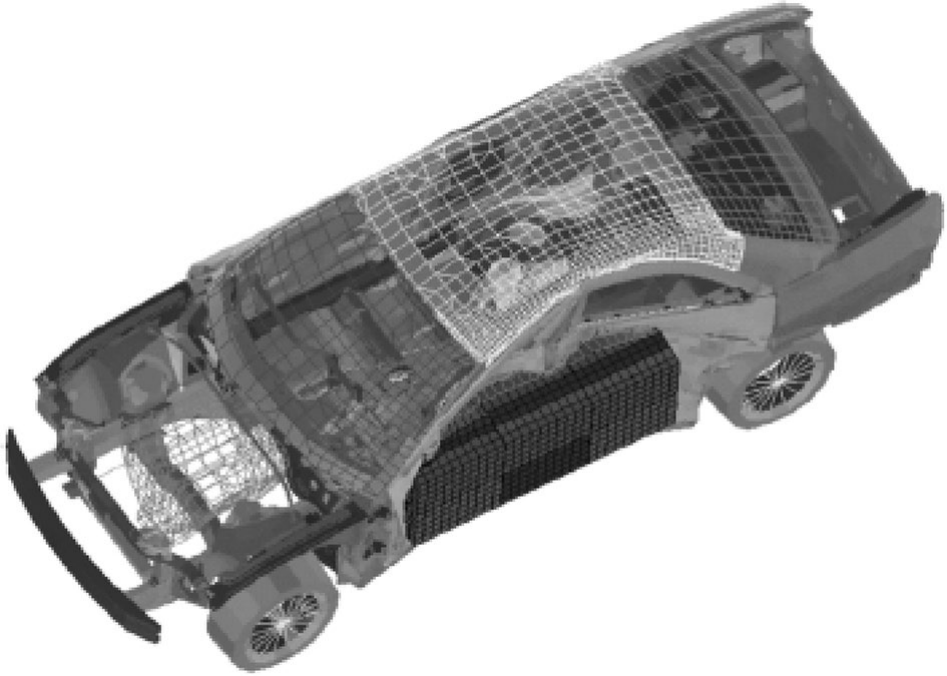
Schematic of car side impact design problem^42^.

The car-side impact design problem, a notorious challenge in engineering, stands out as a complex and time-consuming optimization task, as depicted in Fig. 6. This problem, adaptable to both single-objective and multi-objective formulations, adds layers of intricacy due to its quadratic objective function and explicit models. Specifically, when addressing the single-objective version with the goal of minimizing car weight, the constraints and objective function take on quadratic forms, amplifying the intricacies involved. The thicknesses of the inner floor side (${X}_{3}$) are strategically selected as decision variables to solve the explicit models, and their bounds are dynamically adjusted to accommodate the interconnected variables, adding an additional layer of complexity.

**Figure 7:**
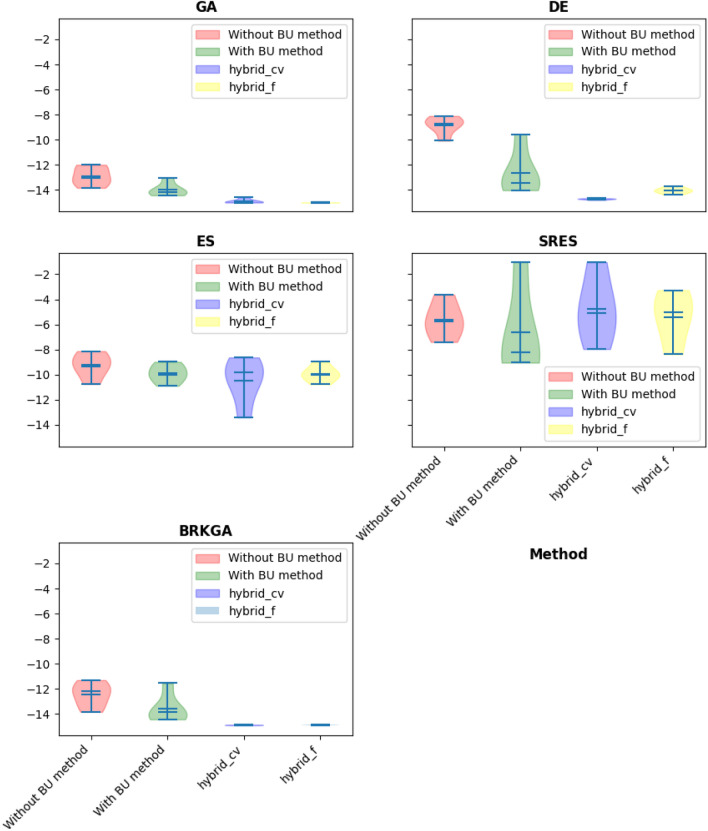
Violin plot of implementation of using different algorithms constraint handling methods (Carside problem).

The results of this formidable optimization challenge are presented in Tables 7, 8, 9 and 10 and Figure 7, offering a quantitative assessment of algorithmic performance. Statistical tests (Tables 8, 9, 10) and visualizations in Figure 7 highlight the superiority of the hybrid methods, particularly when combined with the genetic algorithm (GA). This dominance is established through the comparative analysis, showcasing that the hybrid approach consistently outperforms both the individual methods and, notably, requires fewer function evaluations (FE) to attain the final solution.

**Table 7:** Statistical results of different methods on car side design impact problem.

Algorithm	BU step	Best	Mean	Median	Worst	St. Dev
GA	None^a^	23.889	24.474	24.246	25.295	0.486
	BU	23.538	23.558	23.563	23.568	0.011
	Hybrid-cvtol	**23.522**	**23.551**	**23.557**	**23.566**	0.015
	Hybrid-ftol	23.566	23.714	23.602	24.189	0.238
DE	None	21.920	22.370	22.142	24.389	0.661
	BU	20.797	21.842	21.786	23.128	0.696
	Hybrid-cvtol	**20.750**	21.784	**21.782**	23.067	0.618
	Hybrid-ftol	21.318	**21.714**	23.602	**22.556**	0.238
ES	None	20.432	20.770	20.815	21.168	0.251
	BU	**20.147**	**20.453**	20.540	**20.585**	0.161
	Hybrid-cvtol	**20.147**	20.584	**20.434**	21.386	0.422
	Hybrid-ftol	20.446	20.746	20.727	21.064	0.234
SRES	None	20.518	20.673	20.728	20.760	0.092
	BU	**20.246**	**20.424**	20.436	**20.528**	0.101
	Hybrid-cvtol	20.249	20.425	20.436	**20.528**	0.100
	Hybrid-ftol	20.395	20.477	**20.432**	20.580	0.077
BRKGA	None	**18.165**	**18.904**	**18.90**	**19.572**	0.493
	BU	20.088	20.246	20.202	20.590	0.178
	Hybrid-cvtol	19.631	20.082	20.124	20.639	0.346
	Hybrid-ftol	19.359	20.127	20.20	20.534	0.411

^a^“None” means no BU method or hybrid is used and only feasibility rules as an explicit constraint handling method is worked alone.

Significant values are in bold.

**Table 8:** Summary of p value of the Friedman rank test over all runs.

Algorithm	p-value	Statistic value
GA	0.001	15.00
DE	0.025	9.339
ES	0.162	5.125
SRES	0.0175	10.125
BRKGA	0.020	9.734

**Table 9:** Summary of the p-value of the posthoc_nemenyi_friedman over all runs.

Algorithm	Hybrid-cvtol vs. BU		Hybrid-cvtol vs. Without BU		BU vs. Without BU	
	p-value	h	p-value	h	p-value	h
GA	0.59	~	**0.001**	+	0.06	+
DE	0.72	~	**0.02**	+	0.26	~
ES	0.90	~	0.31	~	0.20	~
SRES	0.87	~	0.20	~	**0.03**	+
BRKGA	0.87	~	0.15		**0.02**	+

Significant values are in bold.

**Table 10:** Summary of the p-value of the posthoc_nemenyi_friedman over all runs (continue).

Algorithm	Hybrid-ftol vs. BU		Hybrid-ftol vs. Without BU		Hybrid-ftol vs. Hybrid-cvtol	
	p-value	h	p-value	h	p-value	h
GA	0.59	~	0.59	~	0.06	~
DE	0.90	~	0.09	~	0.90	~
ES	0.59	~	0.87	~	0.73	~
SRES	0.90	~	**0.03**	+	0.87	~
BRKGA	0.90	~	**0.06**	~	0.90	~

Significant values are in bold.

**Figure 8:**
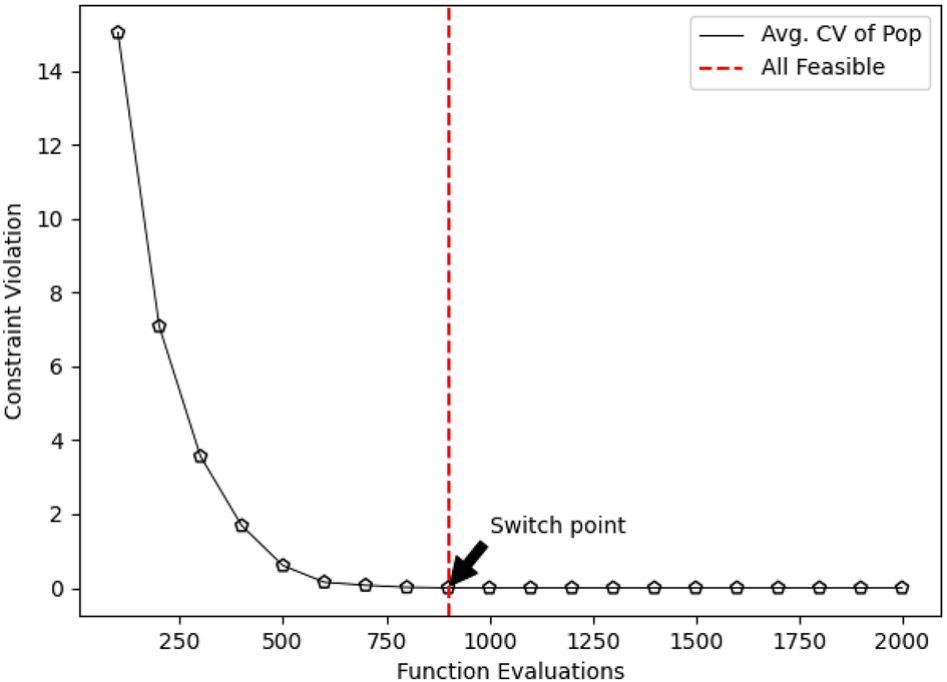
Switch point for GA to normal optimization without the BU method.

Significantly, the examination of the results reveals nuanced differences between the differential evolution (DE) and evolutionary strategy (ES) methods, with the hybrid method showcasing slightly superior outcomes. The incorporation of the BU method, along with the hybrid methods, demonstrates a tangible advantage, resulting in enhanced solution quality. The visual representation of the switching point for GA in Figure 8 provides further clarity on the optimization process. The algorithm strategically transitions from the BU-assisted phase to the standard optimization phase, showcasing an adaptive mechanism designed to optimize the convergence path. However, the study identifies limitations within the context of the car-side impact design problem. While GA, BU, and hybrid methods exhibit notable improvements, the performance of biased random key genetic algorithm (BRKGA) and stochastic ranking evolution strategy (SRES) fails to show enhancement when incorporated into the hybrid framework. This suggests that BRKGA and SRES may not be suitable for addressing the intricacies of this particular problem, underscoring the need for algorithmic adaptability in the face of diverse engineering challenges.

In conclusion, the car-side impact design problem proves to be a formidable optimization challenge, and the hybrid methods, particularly in conjunction with GA, emerge as the most effective approach. The adaptability of the hybrid methods, strategic switching mechanisms, and the incorporation of the BU method collectively contribute to enhanced solution quality and computational efficiency. Nonetheless, the study sheds light on the context-specific nature of algorithmic suitability, emphasizing the importance of tailoring optimization strategies to the unique characteristics of complex engineering problems.

#### Speed reducer

The examination of the speed reducer design problem, featuring one objective function and seven continuous decision variables, has yielded multifaceted insights into the interplay between algorithmic approaches and problem-specific characteristics. With the overarching goal of minimizing the total weight of the speed reducer, the complexity of this task is compounded by a combination of linear and nonlinear constraints. Strategically leveraging three repairing variables—specifically, the face width and shaft diameters (${x}_{1}$, ${x}_{6}$, and ${x}_{7}$)—proves instrumental in managing these constraints effectively.

**Figure 9:**
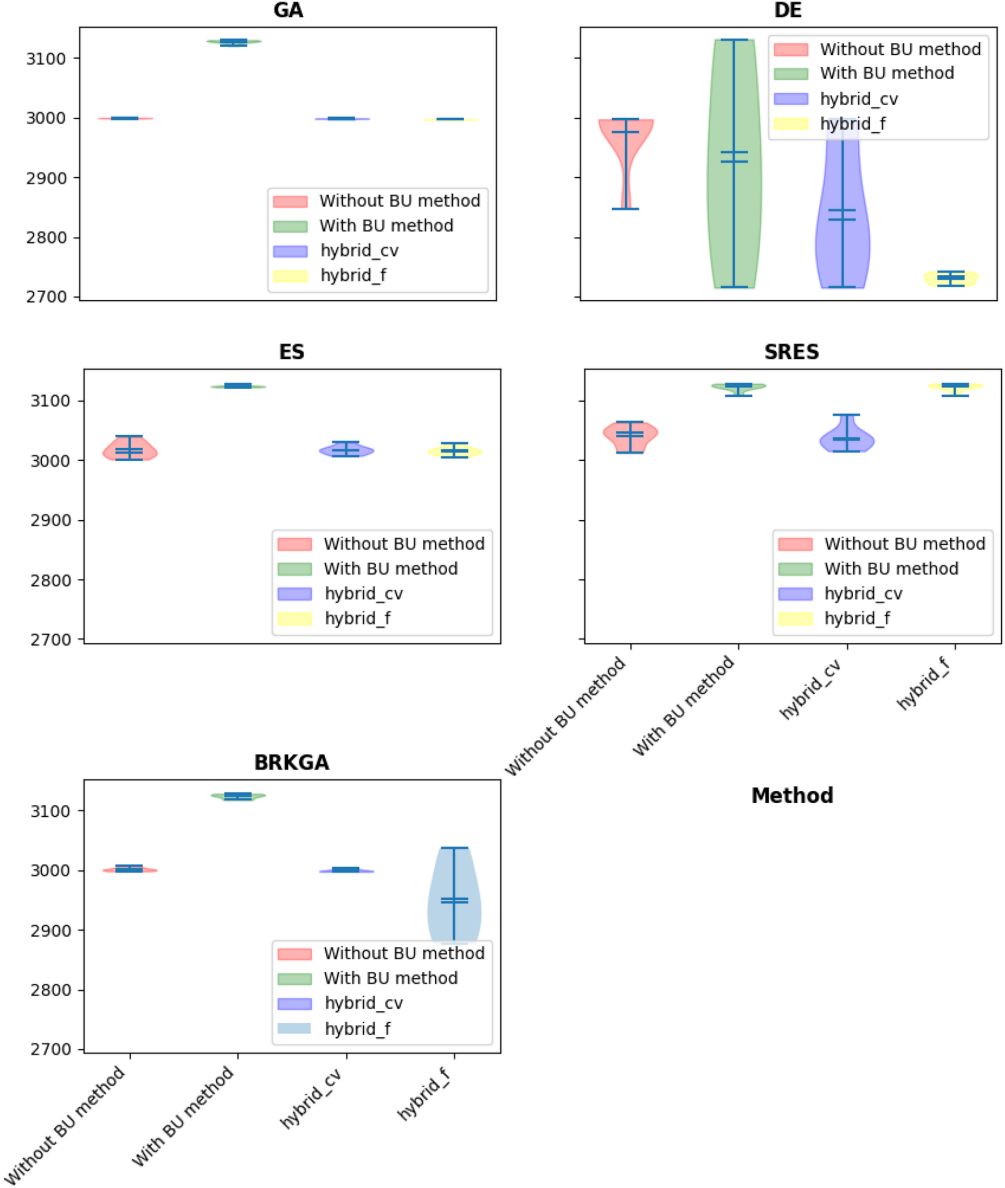
Violin plot of implementation of using different algorithms constraint handling methods (speed reducer problem).

The results, meticulously presented in Tables 11, 12, 13 and 14 and Fig. 9, reveal a consistent trend: the hybrid approaches consistently outperform standalone methods, yielding the best objective function values across all algorithms considered. This dominance suggests that the hybridization process contributes to algorithmic robustness, offering a versatile strategy to navigate the intricate optimization landscape presented by the speed reducer design problem.

**Table 11:** Statistical results of different methods on speed reducer problem.

Algorithm	BU step	Best	Mean	Median	Worst	St. Dev
GA	None^a^	2997.353	2998.827	2998.933	2999.647	0.645
	BU	3119.764	3126.639	3127.78	3129.697	2.792
	Hybrid-cvtol	2997.002	2997.964	2998.023	2998.779	0.539
	Hybrid-ftol	**2996.371**	**2996.422**	**2996.404**	**2996.542**	0.055
DE	None	2847.208	2982.631	2996.641	2997.054	42.848
	BU	2706.172	2900.272	2881.668	3131.199	157.048
	Hybrid-cvtol	**2706.141**	**2838.565**	**2828.955**	2996.729	108.803
	Hybrid-ftol	2718.692	2996.422	2996.404	**2742.44**	0.055
ES	None	3001.0	3023.363	3025.0	3039.00	12.694
	BU	3120.0	3123.727	3123.0	3129.00	2.561
	Hybrid-cvtol	3007.0	3018.000	3018.0	3030.00	6.281
	Hybrid-ftol	**3005**	**3015.428**	**3015**	**3028.00**	6.694
SRES	None	3012.0	3031.272	3025.0	3063.00	17.669
	BU	3108.0	3123.181	3125.0	3128.00	5.236
	Hybrid-cvtol	**3015.0**	**3039.000**	**3038.0**	**3076.00**	17.832
	Hybrid-ftol	3108	3122.57	3125	3128	6.298
BRKGA	None	2997.996	3001.692	3001.084	3006.936	2.761
	BU	3117.466	3123.452	3123.855	3127.798	3.386
	Hybrid-cvtol	2997.312	3000.977	3000.109	**3006.877**	3.254
	Hybrid-ftol	**2875.699**	**2946.35**	**2951.593**	3036.718	56.361

^a^“None” means no BU method or hybrid is used and only feasibility rules as an explicit constraint handling method is worked alone.

Significant values are in bold.

**Table 12:** Summary of the p-value of the Friedman rank test over all runs.

Algorithm	p-value	Statistic value
GA	0.0001	21.0
DE	0.0018	15.000
ES	0.0031	13.799
SRES	0.0003	18.71
BRKGA	0.0031	13.799

**Table 13:** Summary of the p-value of the posthoc_nemenyi_friedman over all runs.

Algorithm	Hybrid-cvtol vs. BU		Hybrid-cvtol vs. Without BU		BU vs. Without BU	
	p-value	h	p-value	h	p-value	h
GA	0.019	+	**0.46**	~	0.46	~
DE	0.29	~	**0.06**	~	0.87	~
ES	0.06	~	0.90	~	0.03	~
SRES	0.014	+	0.90	~	0.026	+
BRKGA	0.03	+	0.90	~	0.06	~

Significant values are in bold.

**Table 14:** Summary of the p-value of the posthoc_nemenyi_friedman over all runs (continue).

Algorithm	Hybrid-ftol vs. BU		Hybrid-ftol vs. Without BU		Hybrid-ftol vs. Hybrid-cvtol	
	p-value	h	p-value	h	p-value	h
GA	0.001	+	0.019	+	0.46	~
DE	0.03	+	0.003	+	0.76	~
ES	0.002	+	0.82	~	0.70	~
SRES	0.90	~	0.02	+	0.014	+
BRKGA	0.002	+	0.70	~	0.82	~

Notably, the algorithms (GA, DE, and BRKGA) exhibit improved performance when integrated into hybrid frameworks, signifying a synergistic effect where the strengths of individual algorithms complement one another. The statistical tests conducted (Tables 12, 13, 14) affirm the significance of these improvements, providing a robust validation of the observed enhancements and instilling confidence in the findings.

A closer examination of GA within the hybrid methods reveals its particular suitability for this problem, consistently outperforming standalone and BU-assisted methods. This positions GA as a robust choice for addressing the complexities inherent in the optimization landscape of the speed reducer design problem.

The incorporation of the BU method consistently leads to a substantial reduction in the number of function evaluations required to reach the final solution. This reduction in computational cost is a valuable contribution, emphasizing the efficiency gains afforded by the BU method, a crucial consideration in engineering optimization scenarios.

Despite the positive outcomes, the study highlights that algorithms like BRKGA and SRES do not exhibit improved performance within the hybrid framework, suggesting their unsuitability for this particular problem. This observation underscores the importance of algorithmic adaptability and tailoring strategies to specific problem characteristics.

In conclusion, the insights gained from the speed reducer design problem analysis underscore the effectiveness of hybrid approaches in enhancing algorithmic performance. The adaptability of GA, efficiency gains with the BU method, and the nuanced trade-offs between algorithms offer valuable guidance for tackling real-world engineering optimization challenges. These findings contribute not only to the understanding of algorithmic behavior in this specific context but also pave the way for broader applications in engineering design optimization.

### Multi-objective optimization problems

The following subsections provide two benchmarks and two real-world optimization problems to assess the effectiveness of the proposed hybrid method for multi-objective optimization problems.

#### Osyczka and Kundu (OSY)

Osyczka and Kundu^44^ introduced the OSY test problem, a widely recognized benchmark featuring two nonlinear objective functions and six linear and nonlinear constraints. As highlighted earlier, the BU method is a central focus of this work, particularly in its ability to directly handle constraints and its subsequent integration with other explicit constraint-handling techniques. The BU approach strategically reduces the feasible search space by altering one or more variables, compelling the algorithm to concentrate on exploring regions that adhere to the defined constraints. For the OSY problem, a specific repairing variable selection strategy is employed, targeting variables ${x}_{1}$, ${x}_{4}$, and ${x}_{6}$ identified as effective for handling all constraints inherent in the problem.

Following the application of the BU approach, NSGA-II is employed to navigate the complex landscape of the OSY problem. NSGA-II, known for identifying viable Pareto fronts, initially achieves an entirely feasible population after 1500 evaluations. In contrast, the coupling of the BU method with the algorithm accelerates this process, achieving a fully feasible population after only 300 evaluations. Subsequently, the optimization process seamlessly transitions to routine optimization without the continued use of the BU method.

**Figure 10:**
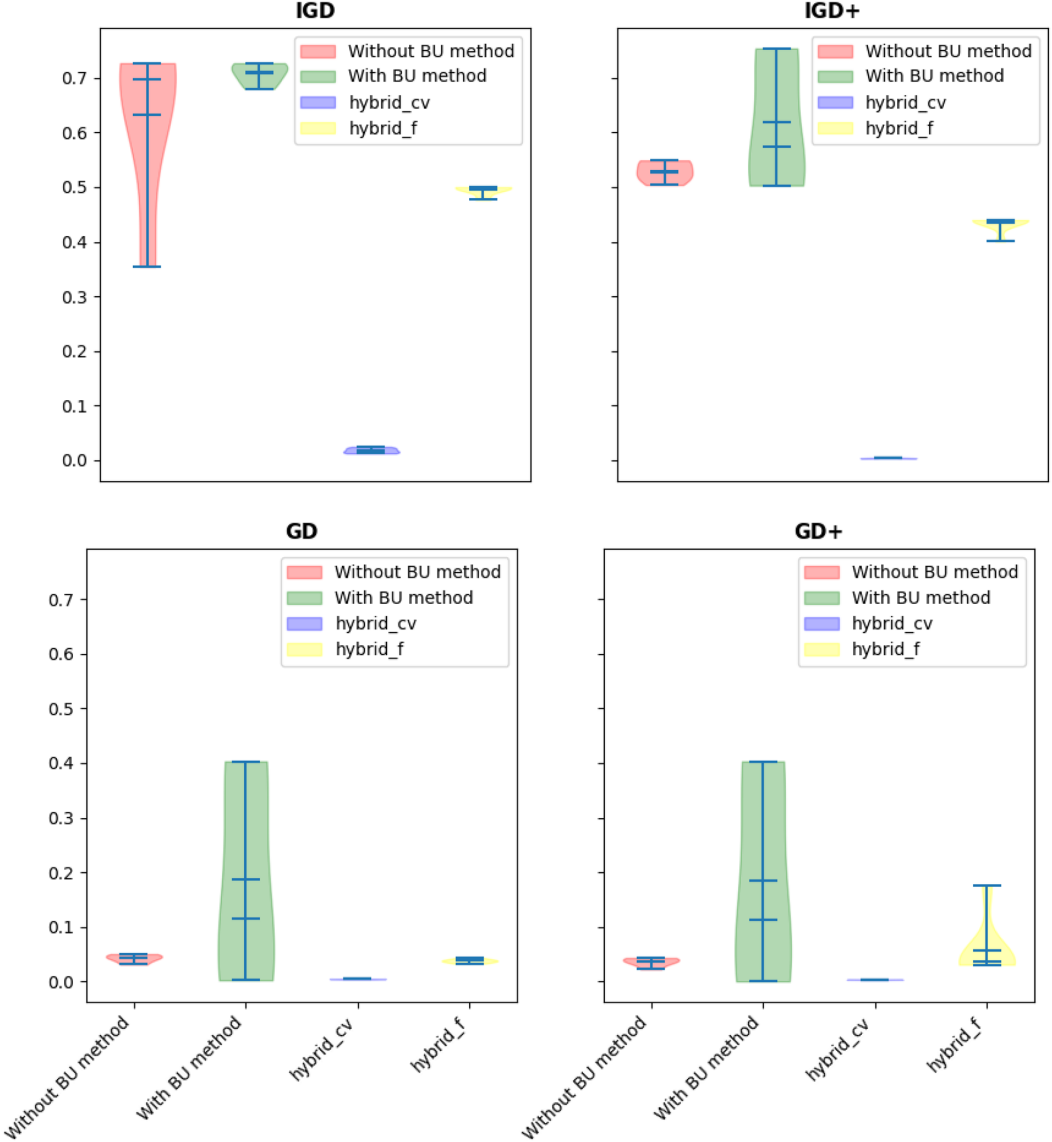
Violin plot of implementation of using different indicators (OSY problem).

The evaluation of performance indicators, presented in Tables 15, 16, 17 and 18 and Fig. 10, offers a comprehensive understanding of the algorithmic performance. It becomes evident that the hybrid methods, particularly when incorporating the BU approach, consistently outperform other methods. Statistical analyses, including the Friedman rank and posthoc tests, validate this observation by demonstrating a significant improvement in all performance indicators (excluding IGD+) when the hybrid approach is implemented.

**Table 15:** Statistical results of different methods on the OSY problem.

Performance indicator	BU step	Best	Mean	Median	Worst	St. Dev
IGD	None^a^	**0.354**	0.652	0.697	0.727	0.12
	BU	0.680	0.712	0.698	0.772	0.032
	Hybrid-cvtol	0.636	0.655	0.651	0.677	0.014
	Hybrid-ftol	0.412	**0.484**	**0.499**	**0.503**	0.030
IGD+	None	0.504	0.520	0.526	0.549	0.013
	BU	0.503	0.573	0.506	0.754	0.107
	Hybrid-cvtol	0.491	0.495	0.495	0.500	0.003
	Hybrid-ftol	**0.400**	**0.433**	**0.439**	**0.439**	0.013
GD	None	0.031	0.042	0.046	0.050	0.007
	BU	**0.002**	0.114	**0.007**	0.403	0.17
	Hybrid-cvtol	0.006	**0.009**	0.010	0.013	0.002
	Hybrid-ftol	0.032	0.057	0.038	0.176	0.048
GD+	None	0.022	0.034	0.037	0.043	0.006
	BU	**0.000**	0.113	**0.006**	0.403	0.172
	Hybrid-cvtol	0.001	**0.004**	0.005	**0.009**	0.002
	Hybrid-ftol	0.030	0.055	0.037	0.175	0.048

^a^“None” means no BU method or hybrid is used and only feasibility rules as an explicit constraint handling method is worked alone.

Significant values are in bold.

**Table 16:** Summary of the p-value of the Friedman rank test over all runs.

Performance indicator	p-value	statistic value
IGD	0.0006	17.22
IGD+	0.0003	18.44
GD	0.0005	17.38
GD+	0.0006	17.26

**Table 17:** Summary of the p-value of the posthoc_nemenyi_friedman over all runs.

Performance indicator	Hybrid-cvtol vs. BU		Hybrid-cvtol vs. Without BU		BU vs. Without BU	
	p-value	h	p-value	h	p-value	h
IGD	0.03	+	0.005	+	0.90	~
IGD+	0.10	~	0.005	+	0.70	~
GD	0.019	+	0.010	+	0.90	~
GD+	0.04	+	0.026	+	0.90	~

**Table 18:** Summary of the p-value of the posthoc_nemenyi_friedman over all runs (continue).

Performance indicator	Hybrid-ftol vs. BU		Hybrid-ftol vs. Without BU		Hybrid-ftol vs. Hybrid-cvtol	
	p-value	h	p-value	h	p-value	h
IGD	0.06	~	0.01	+	0.90	~
IGD+	0.06	~	0.002	+	0.90	~
GD	0.035	+	0.019	+	0.90	~
GD+	0.014	+	0.007	+	0.90	~

Examining Fig. 10 through the lens of a violin plot further illustrates the superiority of the hybrid methods. The best values for all performance indicators, with the exception of IGD, show marked improvement when the BU and hybrid methods are employed. This enhancement in performance underscores the effectiveness of the BU method in combination with the hybrid approach, providing valuable insights into the algorithmic strategies that lead to improved convergence and solution quality for the OSY problem.

In summary, the OSY problem, coupled with the BU method and hybrid approach, serves as a compelling case study. The specific variable selection strategy, the seamless transition between BU-assisted and routine optimization, and the consistent improvement in performance indicators highlight the adaptability and effectiveness of the proposed approach. These findings contribute not only to the optimization of the OSY problem but also offer broader insights into the potential of the BU method and hybridization in addressing complex, constrained optimization scenarios.

#### Bin and Korn Test Problem (BNH)

BNH is a test problem proposed by Binh and Korn^5^, characterized by two conflicting objectives and two constraints, introduces a challenging landscape for optimization. The nonlinear nature of the objectives and constraints adds an additional layer of complexity, requiring sophisticated algorithmic strategies to navigate the intricate solution space effectively. The objectives and constraints may exhibit intricate relationships, posing a considerable challenge to traditional optimization approaches.

**Figure 11:**
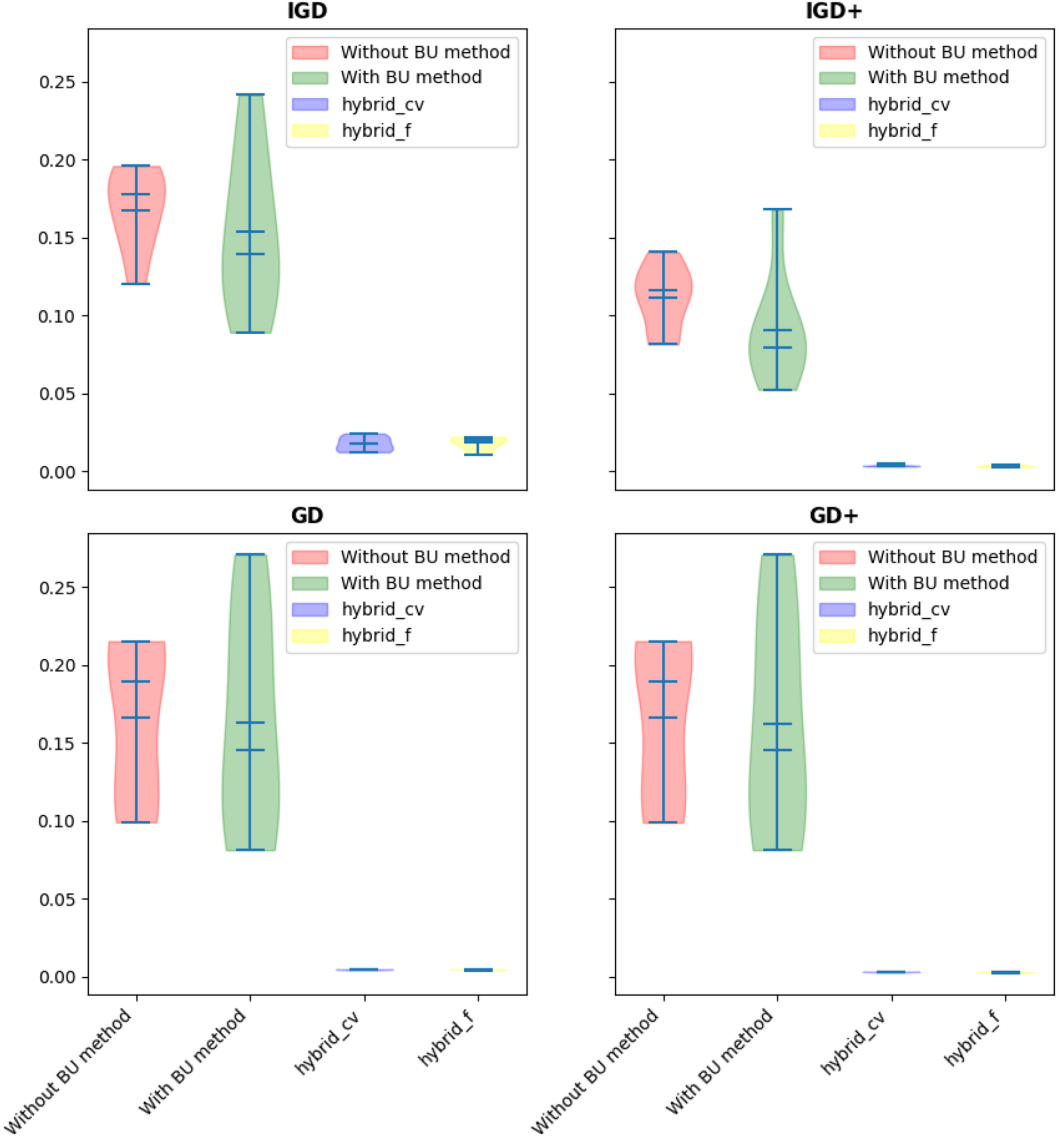
Violin plot of implementation of using different indicators (BNH problem).

In addressing this nonlinear bi-objective optimization problem, the algorithmic focus will be on developing robust strategies that balance the optimization of both objectives while adhering to the imposed constraints. The inherent trade-offs between conflicting objectives necessitate a careful exploration of the Pareto front to identify solutions that represent optimal compromises. This problem involves two continuous variables, ${x}_{1}$ and ${x}_{2}$, which can be strategically chosen as repairing variables. The findings presented in Tables 19, 20, 21, 22, 23, 24 and 25 and Fig. 11 illuminate a substantial disparity between the hybrid method and alternative approaches. Particularly noteworthy is the superior performance of the hybrid method, especially when objective function tolerance is considered, showcasing noteworthy enhancements over other methods across various cases. The results unequivocally demonstrate the hybrid approaches' consistent outperformance in tackling this bi-objective optimization problem characterized by nonlinear constraints and objective functions. Furthermore, the performance indicator values associated with the hybrid approaches are significantly smaller compared to their counterparts, underscoring the effectiveness of the proposed methodology in achieving superior convergence and solution quality. These insights not only contribute to the optimization of the BNH problem but also offer valuable considerations for addressing the nuances of nonlinear bi-objective optimization challenges across diverse applications.

**Table 19:** Statistical results of different methods on the BNH problem.

Performance indicator	BU step	Best	Mean	Median	Worst	St. Dev
IGD	None^a^	0.0423	0.0803	0.0834	0.1281	0.0253
	BU	0.0255	0.0535	0.0426	0.0896	0.0272
	Hybrid-cvtol	0.0123	0.0289	0.0209	0.0660	0.0172
	Hybrid-ftol	**0.0110**	**0.0183**	**0.0200**	**0.0222**	0.0038
IGD+	None	0.0170	0.0268	0.0247	0.0433	0.0084
	BU	0.0112	0.0222	0.0179	0.0419	0.0111
	Hybrid-cvtol	**0.0024**	0.0061	0.0048	0.0143	0.0040
	Hybrid-ftol	0.0029	**0.0035**	**0.0033**	**0.0043**	0.0005
GD	None	0.0045	0.0052	0.0050	0.0065	0.0006
	BU	0.0061	0.0073	0.0066	0.0098	0.0013
	Hybrid-cvtol	0.0044	0.0046	0.0046	0.0049	0.0001
	Hybrid-ftol	**0.0040**	**0.0044**	**0.0046**	**0.0047**	0.0002
GD+	None	0.0029	0.0034	0.0033	0.0048	0.0005
	BU	0.0039	0.0053	0.0049	0.0079	0.0013
	Hybrid-cvtol	0.0026	**0.0027**	**0.0027**	**0.0029**	0.00009
	Hybrid-ftol	**0.0020**	0.0028	0.0030	0.0031	0.0003

Significant values are in bold.

**Table 20:** Summary of the p-value of the Friedman rank test over all runs.

Performance indicator	p-value	Statistic value
IGD	0.0006	17.22
IGD+	0.0003	18.44
GD	0.0005	17.38
GD+	0.0006	17.26

**Table 21:** Summary of the p-value of the posthoc_nemenyi_friedman over all runs.

Performance indicator	Hybrid-cvtol vs. BU		Hybrid-cvtol vs. Without BU		BU vs. Without BU	
	p-value	h^a^	p-value	h	p-value	h
IGD	0.244362	~	0.003837	+	0.244362	~
IGD+	0.244362	~	0.003837	+	0.244362	~
GD	0.001488	+	0.376245	~	0.082380	~
GD+	0.001000	+	0.147296	~	0.147296	~

^a^Posthoc_nemenyi_friedman: +, ~, and – presents the first method performs statistically significantly better, equal, and worse than the second approach.

**Table 22:** Summary of the p-value of the posthoc_nemenyi_friedman over all runs- IGD value comparison.

Method	Hybrid-cvtol	Hybrid-ftol	BU	Without BU
Hybrid-cvtol	–	0.9000	0.0358	0.0051
Hybrid-ftol	0.9000	–	0.06243	0.01026
BU	0.0358	0.06243	–	0.9000
Without BU	0.0051	0.01026	0.9000	–

**Table 23:** Summary of the p-value of the posthoc_nemenyi_friedman over all runs-IGD+value comparison.

Method	Hybrid-cvtol	Hybrid-ftol	BU	Without BU
Hybrid-cvtol	–	0.900000	0.103359	0.005121
Hybrid-ftol	0.900000	–	0.062433	0.002444
BU	0.103359	0.062433	–	0.704147
Without BU	0.005121	0.002444	0.704147	-

**Table 24:** Summary of the p-value of the posthoc_nemenyi_friedman over all runs- GD value comparison.

Method	Hybrid-cvtol	Hybrid-ftol	BU	Without BU
Hybrid-cvtol	–	0.9000	0.019641	0.010266
Hybrid-ftol	0.9000	–	0.035858	0.019641
BU	0.019641	0.035858	–	0.9000
Without BU	0.010266	0.019641	0.9000	–

**Table 25:** Summary of the p-value of the posthoc_nemenyi_friedman over all runs- GD+value comparison.

Method	Hybrid-cvtol	Hybrid-ftol	BU	Without BU
Hybrid-cvtol	–	0.9000	0.047574	0.026690
Hybrid-ftol	0.9000	–	0.014282	0.007293
BU	0.047574	0.014282	–	0.9000
Without BU	0.026690	0.007293	0.9000	–

#### Welded beam design problem

The welded beam fabrication is a well-known engineering optimization benchmark problem^48^ that examines the trade-off between the strength and cost of a beam. The problem includes four constraints (Eqs. 32–35) and four design variables, which are:

- ${x}_{1}$ = *h*, the thickness of the welds
- ${x}_{2}$ = *l*, the length of the welds
- ${x}_{3}$ = *t*, the height of the beam
- ${x}_{4}$ = *b*, the width of the beam

The constraints are:

33 $${g}_{1}=\left(\frac{1}{{t}_{max}}\right)\times \left(t-{t}_{max}\right)\le 0$$

34 $${g}_{2}=\left(\frac{1}{{s}_{max}}\right)\times \left(s-{s}_{max}\right)\le 0$$

35 $${g}_{3}=\left(\frac{1}{5-0.125}\right)\times \left({x}_{1}-{x}_{4}\right)\le 0$$

36 $${g}_{4}=\left(\frac{1}{P}\right)\times (P-{P}_{c})\le 0$$

where ${t}_{max}=13600, {s}_{max}=30000, P=6000, {P}_{c}=64746.022 \times \left(1 - 0.0282346 \times {x}_{3}\right)\times {x}_{3}\times {x}_{4}^{3}, s=6\times P\times 14/({x}_{4}\times {x}_{3}^{2})$. The two objective functions are to minimize the fabrication cost of the beam and minimize the deflection of the end of the beam:

37 $${f}_{1}=1.10471 \times {x}_{1}^{2}\times {x}_{2}+0.04811\times {x}_{3}\times {x}_{4}\times (14.0+{x}_{2}))$$

38 $${f}_{2}=2.1952/({x}_{4}\times {x}_{3}^{3})$$

The four variables of this problem include: (1) thickness of the weld, (2) welded joint's length, (3) beam's width, and (4) beam's thickness. Using the three strategies, NSGA-II was applied to this problem, and fitness values were evaluated.

**Figure 12:**
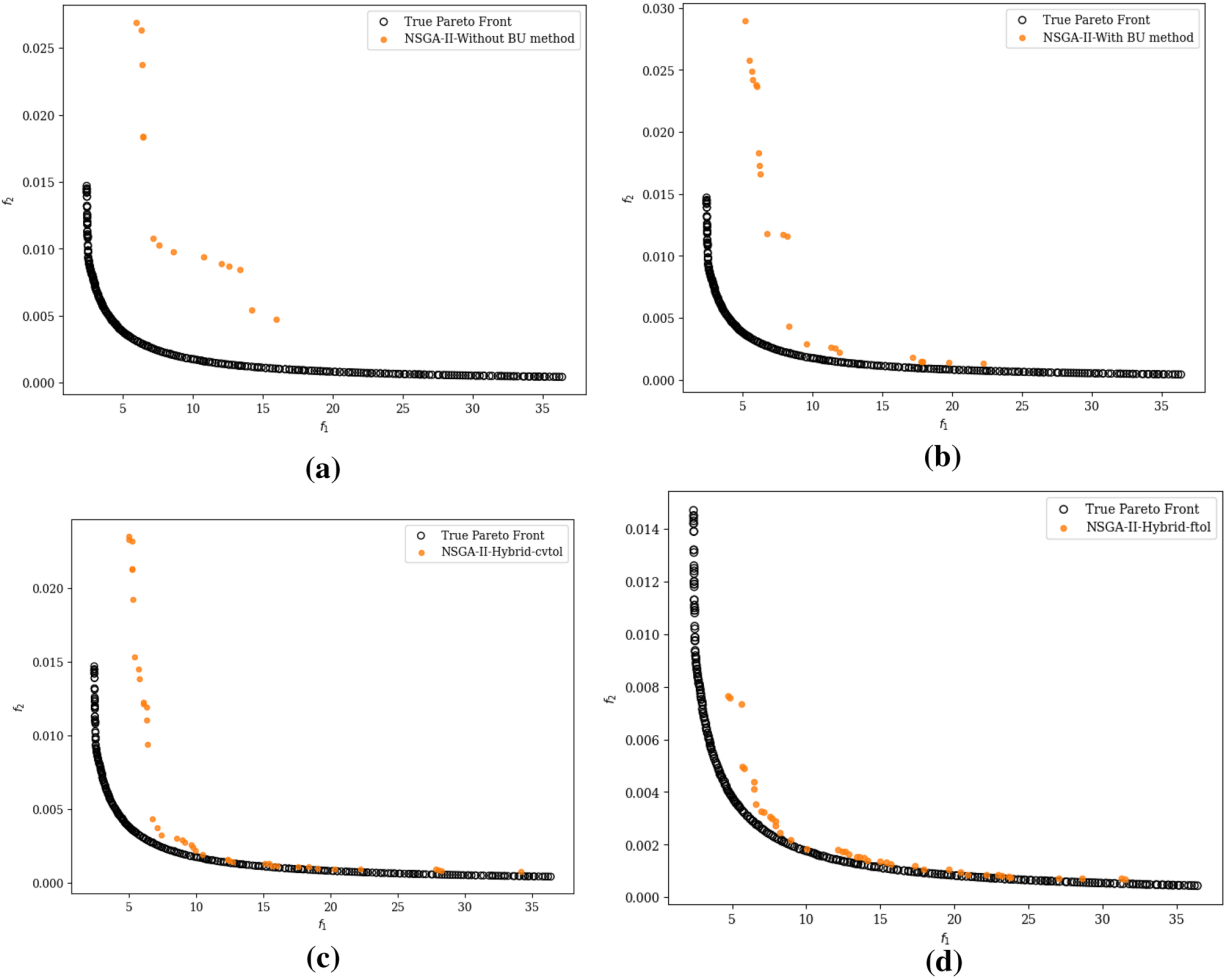
Pareto-optimal fronts obtained for the welded beam design problem for both BU and without BU compared with the true Pareto-optimal front (Generation 20).

The exploration of sampling and crossover operators in EAs stands as a critical endeavor, influencing the convergence speed and efficacy of these algorithms, particularly in the context of noisy optimization. In the context of the Welded Beam Design problem, where the impact of noise is significant, the experimentation involved diverse operators, including real random sampling, Latin hypercube sampling, and two distinct crossovers: real-SBX crossover and uniform crossover. Four performance indicators, namely GD, GD+ , IGD, and IGD+, were meticulously evaluated against these operators to glean insights into their comparative effectiveness. The compelling findings, detailed in Tables 26, 27, 28, 29, 30, 31 and 32 and illustrated in Fig. 12, underscore the superiority of the hybrid approach over the two alternative methods.

**Table 26:** Statistical results of different methods on the welded beam design problem (sampling).

Performance indicator	Sampling											
	Real_lhs						Real_random					
	BU step	Best	Mean	Median	Worst	St. Dev	BU step	Best	Mean	Median	Worst	St. Dev
IGD	None^a^	0.12	0.15	0.147	0.179	0.021	None	0.12	0.167	0.178	0.19	0.024
	BU	0.05	0.15	0.161	0.239	0.051	BU	0.08	0.153	0.139	0.24	0.048
	Hybrid-cvtol	**0.01**	**0.03**	**0.021**	0.089	0.028	Hybrid-cvtol	**0.01**	**0.033**	**0.034**	**0.05**	0.010
	Hybrid-ftol	0.044	0.058	0.058	**0.069**	0.009	Hybrid-ftol	0.028	0.057	0.061	0.084	0.0190
IGD+	None	0.05	0.08	0.071	0.119	0.023	None	0.08	0.111	0.116	0.14	0.019
	BU	0.03	0.08	0.070	0.138	0.034	BU	0.05	0.090	0.079	0.16	0.034
	Hybrid-cvtol	**0.00**	**0.00**	**0.003**	**0.016**	0.004	Hybrid-cvtol	**0.00**	**0.005**	**0.004**	**0.00**	0.002
	Hybrid-ftol	0.015	0.018	0.018	0.020	0.001	Hybrid-ftol	0.012	0.018	0.018	0.27	0.004
GD	None	0.05	0.11	0.113	0.196	0.037	None	0.09	0.166	0.189	0.21	0.048
	BU	0.02	0.13	0.128	0.282	0.071	BU	0.08	0.162	0.145	0.27	0.067
	Hybrid-cvtol	**0.00**	**0.00**	**0.002**	**0.004**	0.000	Hybrid-cvtol	**0.00**	**0.002**	**0.002**	**0.00**	0.001
	Hybrid-ftol	0.004	0.008	0.008	0.011	0.002	Hybrid-ftol	0.004	0.008	0.005	0.030	0.008
GD+	None	0.05	0.11	0.113	0.196	0.037	None	0.09	0.166	0.189	0.21	0.048
	BU	0.02	0.13	0.128	0.282	0.071	BU	0.08	0.162	0.145	0.27	0.067
	Hybrid-cvtol	**0.00**	**0.00**	**0.001**	**0.003**	0.000	Hybrid-cvtol	**0.00**	**0.001**	**0.001**	**0.00**	0.000
	Hybrid-ftol	0.001	0.007	0.007	0.012	0.004	Hybrid-ftol	0.003	0.008	0.005	0.030	0.009

^a^“None” means no BU method or hybrid is used and only feasibility rules as an explicit constraint handling method is worked alone.

Significant values are in bold.

**Table 27:** Statistical results of different methods on the welded beam design problem (crossover).

Performance indicator	Crossover											
	Uniform						real_sbx					
	BU step	Best	Mean	Median	Worst	St. Dev	BU step	Best	Mean	Median	Worst	St. Dev
IGD	None	0.140	0.166	0.167	0.191	0.017	None	0.080	0.144	0.151	0.197	0.035
	BU	0.080	0.126	0.127	0.192	0.033	BU	0.136	0.167	0.167	0.186	0.015
	Hybrid-cvtol	**0.006**	**0.050**	**0.055**	0.095	0.027	Hybrid-cvtol	**0.009**	**0.032**	**0.028**	0.074	0.022
	Hybrid-ftol	0.032	0.052	0.051	**0.067**	0.012	Hybrid-ftol	0.017	0.033	0.033	**0.046**	0.010
IGD+	None	0.072	0.092	0.096	0.116	0.015	None	0.036	0.071	0.069	0.108	0.025
	BU	0.035	0.071	0.077	0.104	0.023	BU	0.058	0.090	0.088	0.135	0.028
	Hybrid-cvtol	**0.002**	**0.008**	0.009	0.017	0.004	Hybrid-cvtol	**0.001**	**0.005**	**0.005**	0.007	0.002
	Hybrid-ftol	0.005	**0.008**	**0.008**	**0.010**	0.001	Hybrid-ftol	0.003	**0.005**	**0.005**	**0.006**	0.001
GD	None	0.086	0.154	0.176	0.21	0.042	None	0.030	0.102	0.086	0.185	0.053
	BU	0.042	0.146	0.123	0.311	0.081	BU	0.062	0.194	0.189	0.410	0.099
	Hybrid-cvtol	**0.001**	**0.002**	**0.002**	0.005	0.001	Hybrid-cvtol	**0.001**	**0.002**	**0.002**	0.003	0.000
	Hybrid-ftol	**0.001**	**0.002**	**0.002**	**0.002**	0.000	Hybrid-ftol	**0.001**	**0.002**	**0.002**	**0.002**	0.000
GD+	None	0.086	0.154	0.176	0.21	0.042	None	0.030	0.102	0.086	0.185	0.053
	BU	0.042	0.146	0.123	0.311	0.081	BU	0.062	0.194	0.189	0.410	0.099
	Hybrid-cvtol	**0.000**	**0.001**	**0.001**	0.004	0.001	Hybrid-cvtol	**0.000**	**0.001**	**0.001**	0.002	0.000
	Hybrid-ftol	**0.000**	**0.001**	**0.001**	**0.001**	0.0004	Hybrid-ftol	**0.000**	**0.001**	**0.001**	**0.001**	0.000

Significant values are in bold.

**Table 28:** Summary of the p-value of the Friedman rank test over all runs.

Perofrmance indicator	Sampling				Crossover			
	Real_lhs		Real_random		Uniform		real_sbx	
	p-value	Statistic value	p-value	STATISTIC value	p-value	statistic value	p-value	Statistic value
IGD	0.16	10.23	0.01	11.09	0.01	9.89	0.01	10.20
IGD+	0.01	11.15	0.03	8.39	0.01	11.15	0.009	11.36
GD	0.01	11.09	0.01	11.09	0.01	10.78	0.008	11.75
GD+	0.01	11.09	0.1	11.09	0.01	10.78	0.008	11.75

**Table 29:** Summary of the p-value of the posthoc_nemenyi_friedman test evaluated over all runs (sampling).

Performance indicator	Sampling											
	Real_lhs						Real_random					
	Hybrid-cvtol vs. BU		Hybrid-cvtol vs. Without BU		BU vs. Without BU		Hybrid-cvtol vs. BU		Hybrid-cvtol vs. Without BU		BU vs. Without BU	
	p-value	h^a^	p-value	h	p-value	h	p-value	h	p-value	h	p-value	h
IGD	0.04	+	0.09	~	0.90	~	0.06	~	0.01	+	0.90	~
IGD+	0.04	+	0.02	+	0.90	~	0.12	~	0.03	+	0.90	~
GD	0.01	+	0.06	~	0.90	~	0.06	~	0.01	+	0.90	~
GD+	0.01	+	0.06	~	0.90	~	0.06	~	0.01	+	0.90	~

^a^Posthoc_nemenyi_friedman: +, ~, and – presents the first method performs statistically significantly better, equal, and worse than the second approach.

**Table 30:** Summary of the p-value of the posthoc_nemenyi_friedman test evaluated over all runs (sampling-Continue).

Performance indicator	Sampling											
	Real_lhs						Real_random					
	Hybrid-ftol vs. BU		Hybrid-ftol vs. Without BU		Hybrid-ftol vs. Hybrid-cvtol		Hybrid-ftol vs. BU		Hybrid-ftol vs. Without BU		Hybrid-ftol vs. Hybrid-cvtol	
	p-value	h^a^	p-value	h	p-value	h	p-value	h	p-value	h	p-value	h
IGD	0.04	+	0.09	~	0.90	~	0.51	~	0.22	~	0.67	~
IGD+	0.43	~	0.28	~	0.67	~	0.90	~	0.67	~	0.35	~
GD	0.22	~	0.51	~	0.67	~	0.51	~	0.22	~	0.67	~
GD+	0.22	~	0.51	~	0.67	~	0.51	~	0.22	~	0.67	~

^a^Posthoc_nemenyi_friedman: +, ~, and – presents the first method performs statistically significantly better, equal, and worse than the second approach.

**Table 31:** Summary of p value of the posthoc_nemenyi_friedmantest evaluated over all runs (crossover).

Performance indicator	Crossover											
	Uniform						Real_sbx					
	Hybrid-cvtol vs. BU		Hybrid-cvtol vs. Without BU		BU vs. Without BU		Hybrid-cvtol vs. BU		Hybrid-cvtol vs. Without BU		BU vs. Without BU	
	p-value	h^a^	p-value	h	p-value	h	p-value	h	p-value	h	p-value	h
IGD	0.22	~	0.06	~	0.90	~	0.03	+	0.12	~	0.90	~
IGD+	0.43	~	0.04	+	0.67	~	0.03	+	0.35	~	0.67	~
GD	0.28	~	0.09	~	0.90	~	0.04	+	0.43	~	0.67	~
GD+	0.28	~	0.09	~	0.90	~	0.04	+	0.43	~	0.67	~

^a^Posthoc_nemenyi_friedman: +, ~, and – presents the first method performs statistically significantly better, equal, and worse than the second approach.

**Table 32:** Summary of p value of the posthoc_nemenyi_friedmantest evaluated over all runs (crossover-Continue).

Performance indicator	Crossover											
	Uniform						real_sbx					
	Hybrid-ftol vs. BU		Hybrid-ftol vs. Without BU		Hybrid-ftol vs. Hybrid-cvtol		Hybrid-ftol vs. BU		Hybrid-ftol vs. Without BU		Hybrid-ftol vs. Hybrid-cvtol	
	p-value	h^a^	p-value	h	p-value	h	p-value	h	p-value	h	p-value	h
IGD	0.22	~	0.06	~	0.90	~	0.12	~	0.35	~	0.90	~
IGD+	0.28	~	0.02	+	0.90	~	0.03	+	0.35	~	0.67	~
GD	0.16	~	0.04	+	0.90	~	0.02	+	0.28	~	0.90	~
GD+	0.16	~	0.04	+	0.90	~	0.02	+	0.28	~	0.90	~

^a^Posthoc_nemenyi_friedman: +, ~, and – presents the first method performs statistically significantly better, equal, and worse than the second approach.

Examining the Pareto optimal solutions generated by the hybrid methods across different generations reveals a consistent outperformance, particularly noteworthy when considering constraint violations as tolerance with real random sampling. This suggests that the hybrid approach, when coupled with appropriate operators, significantly improves the optimization process for the Welded Beam Design problem. Notably, the hybrid method's effectiveness is evident with Latin hypercube sampling, showcasing its compatibility and superior performance within this optimization context. Furthermore, the hybrid method incorporating objective function tolerance using uniform crossover emerges as a robust option, outperforming optimization without the BU approach.

In contrast to initial expectations regarding the BU approach as a constraint handling technique, the results indicate that it may not be the optimal choice for this specific nonlinear bi-objective model. However, the hybrid approaches, through strategic integration of various operators, demonstrate a substantial enhancement in performance. This nuanced observation highlights the importance of selecting appropriate operators within hybrid approaches, suggesting that the effectiveness of the BU method can be context-dependent.

**Figure 13:**
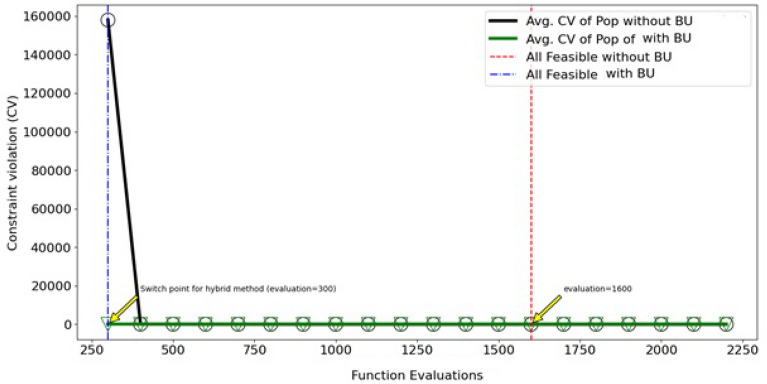
Constraint violation evaluated by NSGA-II (convergence of the whole population to feasible solutions).

The statistical analyses, including the Friedman rank test and the Posthoc_Nemenyi_Friedman, contribute to the robustness of the findings. These tests not only affirm the significant differences between the hybrid approach and the two other methods but also reinforce the superiority of the proposed hybrid method for solving the Welded Beam Design problem. The identification of the switching point for the second phase of the optimization process at evaluation 300 further exemplifies the efficiency of the hybrid approach compared to the without BU approach, where the feasible region is reached at evaluation 1600 (Fig. 13).

In conclusion, the investigation into sampling and crossover operators, coupled with the nuanced insights from performance indicators and statistical analyses, positions the hybrid approach as the preferred strategy for solving the Welded Beam Design problem. The adaptability of the hybrid method, evident in its performance across various operators, reinforces its efficacy in addressing the challenges posed by the specific characteristics of this nonlinear bi-objective optimization scenario.

#### Cantilevered stepped beam design problem

**Figure 14:**
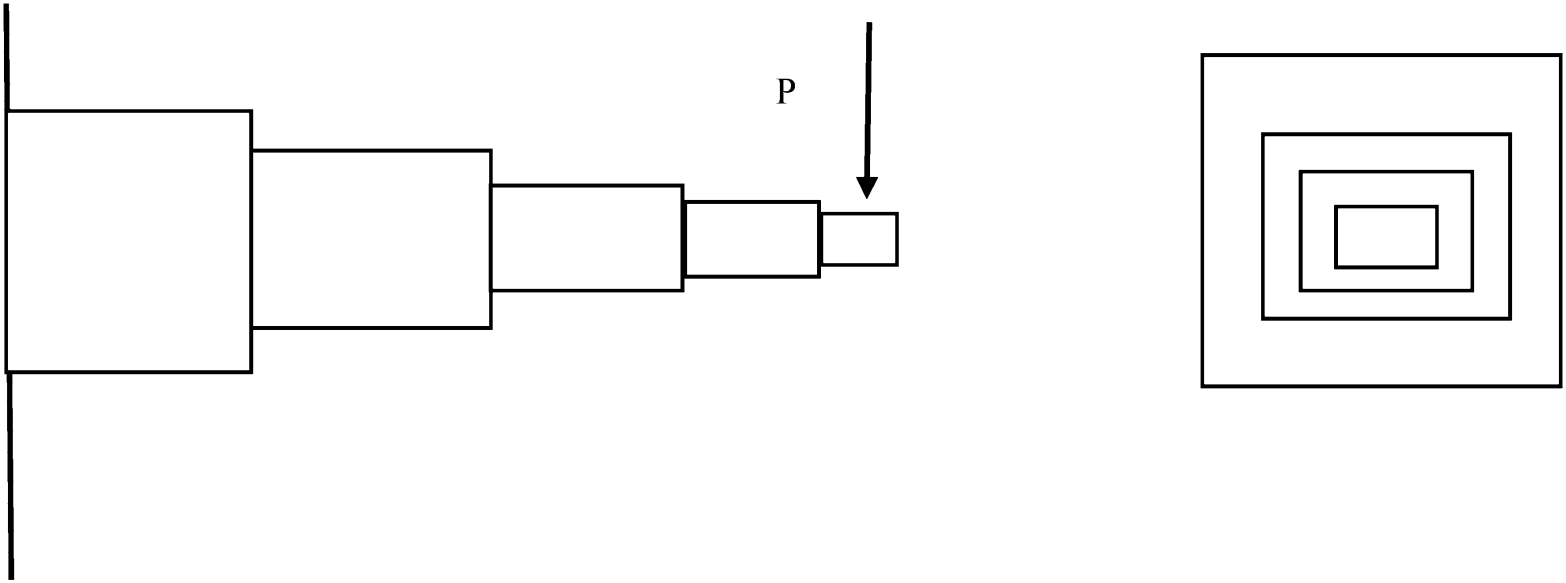
Schematic of the stepped beam design problem.

**Figure 15:**
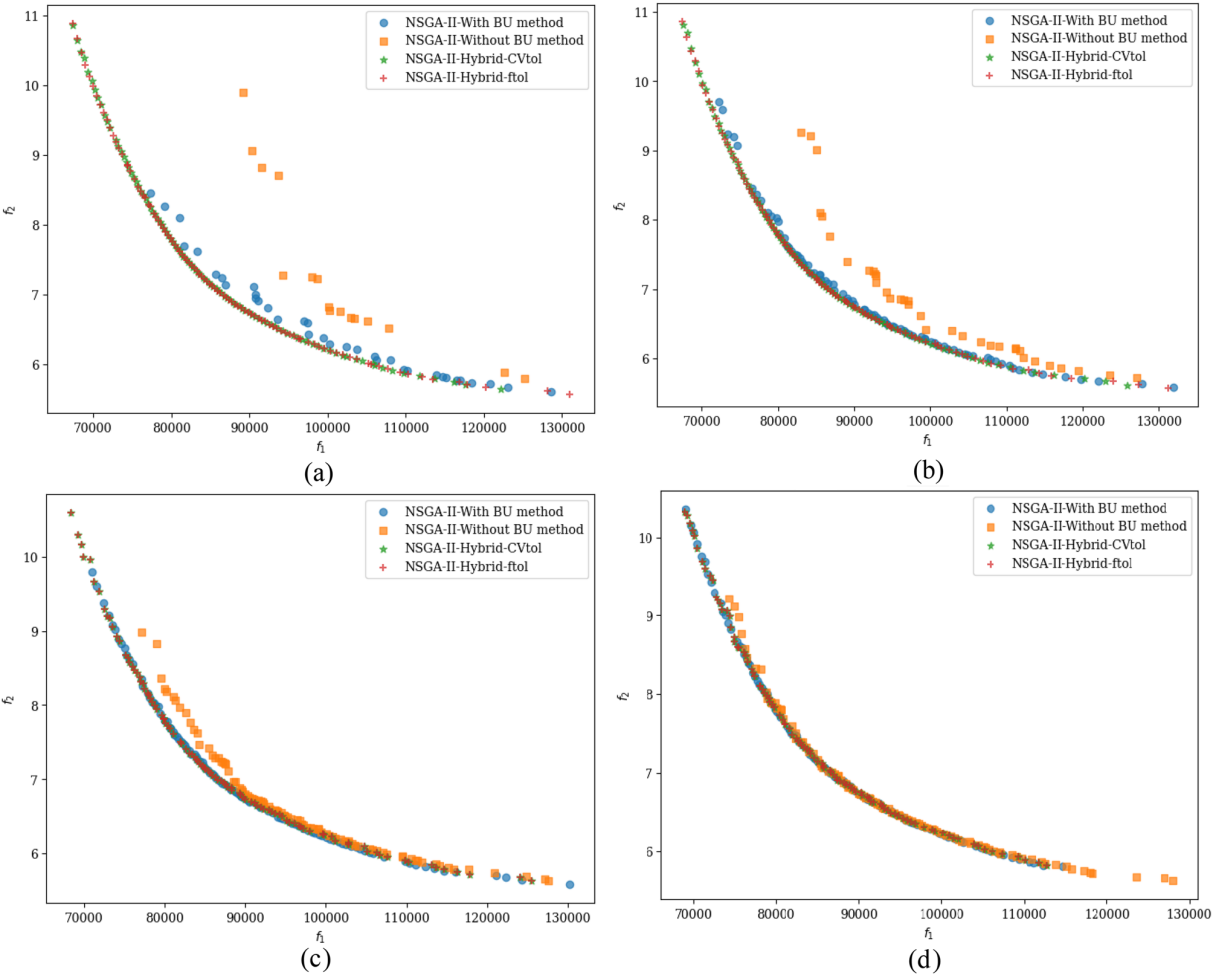
Pareto solutions found by NSGA-II by different approaches and generations.

The cantilevered stepped design problem is an example of large-scale size problem. In the cantilevered beam design problem, the stepped cantilever beam must be able to carry a prescribed end load^46^. This problem is originally a single-objective optimization problem considering minimizing the beam volume; however, this study has extended the original problem and added one more objective function, which minimize end deflection subject to various engineering design variables (see Appendix). The beam supports the given load, P, at a fixed distance L from the support. Besides, designers of the beam can vary the width (${b}_{i}$) and height (${h}_{i}$) of each section (Fig. 14). The problem is highly constraint and large-dimensional; however, for this example five segments (N = 5) including 10 dimensions (10 constraints) are considered. In the pursuit of solving both the single- and multi-objective versions of this intricate problem, repairing variables ${x}_{2}$, ${x}_{4}, {x}_{6,}$ ${x}_{8},$ and ${x}_{10}$ have been identified. The application of the BU method to this constrained nonlinear optimization problem is showcased, providing a valuable demonstration of the BU method's utility. Figure 15a–d visually presents the optimal solutions discovered by NSGA-II employing different methods across various generations.

**Figure 16:**
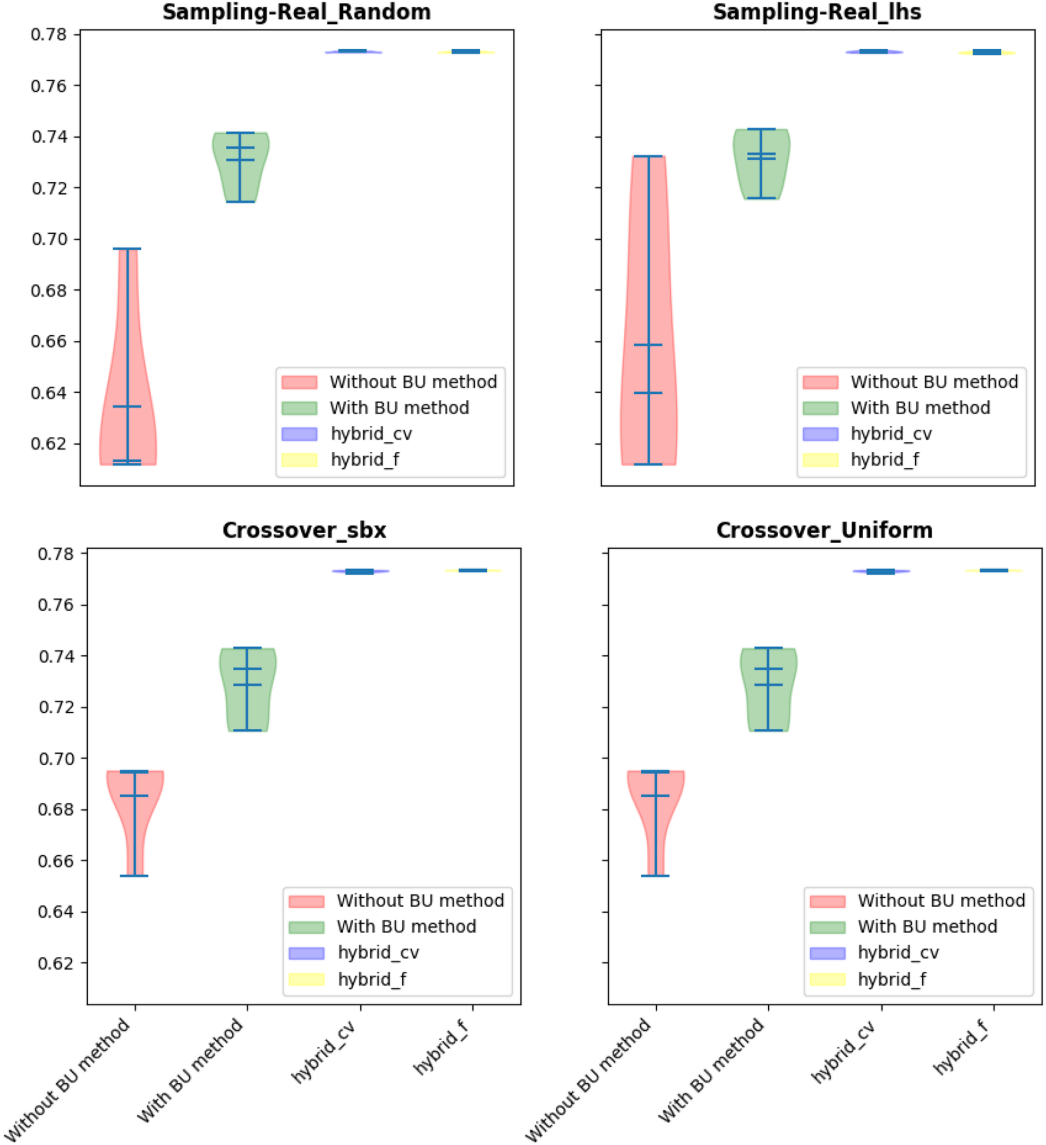
Violin plot of implementation of using different methods (Stepped beam design problem problem).

To assess the performance of the proposed approaches, the hypervolume (HV) indicator is utilized, offering a comprehensive measure of the quality of solutions (Tables 33, 34). The results, detailed in Tables 35, 36, 37, 38 and 39 and visualized in Fig. 16, unequivocally demonstrate the efficacy of the hybrid approaches in enhancing solution quality. This improvement is particularly noteworthy in the case of Latin hypercube sampling and simulated binary crossover (SBX), where the hybrid approaches exhibit a significant enhancement in solution quality.

**Table 33:** Results of different methods on the cantilevered stepped design problem (HV values-sampling).

Algorithm	Sampling											
	Real_random						Real_lhs					
	BU step	Best	Mean	Median	Worst	St. Dev	BU step	Best	Mean	Median	Worst	St. Dev
NSGA-II	None^a^	0.6960	0.6344	0.6131	0.6118	0.0325	None	0.7324	0.6585	0.6396	0.6118	0.0478
	BU	0.7415	0.7309	0.7355	0.7144	0.0102	BU	0.7428	0.7313	0.7329	0.7156	0.0103
	Hybrid-cvtol	**0.7734**	**0.7731**	**0.7730**	**0.7729**	0.0002	Hybrid-cvtol	**0.7737**	**0.7731**	**0.7731**	**0.7727**	0.0003
	Hybrid-ftol	**0.7734**	0.7730	0.7729	0.7728	0.0002	Hybrid-ftol	**0.7737**	0.7729	0.7727	0.7723	0.0004

^a^“None” means no BU method or hybrid is used and only feasibility rules as an explicit constraint handling method is worked alone.

Significant values are in bold.

**Table 34:** Results of different methods on the the cantilevered stepped design problem (HV values-crossover).

Algorithm	Crossover											
	Real_sbx						Uniform					
	BU step	Best	Mean	Median	Worst	St. Dev	BU step	Best	Mean	Median	Worst	St. Dev
NSGA-II	None^a^	0.6544	0.6243	0.6396	0.5762	0.0277	None	0.6950	0.6849	0.6941	0.6539	0.0157
	BU	0.7428	0.7315	0.7347	0.7156	0.0089	BU	0.7428	0.7285	0.7347	0.7106	0.0129
	Hybrid-cvtol	**0.7737**	**0.7733**	**0.7733**	0.7727	0.0003	Hybrid-cvtol	0.7733	0.7728	0.7730	0.7721	0.0004
	Hybrid-ftol	**0.7737**	0.7732	**0.7733**	**0.7729**	0.0002	Hybrid-ftol	**0.7734**	**0.7732**	**0.7732**	**0.7730**	0.0001

^a^“None” means no BU method or hybrid is used and only feasibility rules as an explicit constraint handling method is worked alone.

Significant values are in bold.

**Table 35:** Summary of the p-value of the Friedman rank test over all runs.

Algorithm	Sampling				Crossover			
	Real_random		Real_lhs		real_sbx		Uniform	
	p-value	Statistic value	p-value	Statistic value	p-value	Statistic value	p-value	Statistic value
NSGA-II	0.002	14.61	0.003	13.77	0.002	14.03	0.002	14.03

**Table 36:** Summary of the p-value of the posthoc_nemenyi_friedman test evaluated over all runs (sampling).

Algorithm	Sampling											
	Real_lhs						Real_random					
	Hybrid-cvtol vs. BU		Hybrid-cvtol vs. Without BU		BU vs. Without BU		Hybrid-cvtol vs. BU		Hybrid-cvtol vs. Without BU		BU vs. Without BU	
	p-value	h^a^	p-value	h	p-value	h	p-value	h	p-value	h	p-value	h
NSGA-II	0.16	~	0.008	+	0.67	~	0.16	~	0.008	+	0.67	~

^a^Posthoc_nemenyi_friedman: +, ~, and – presents the first method performs statistically significantly better, equal, and worse than the second approach.

**Table 37:** Summary of the p-value of the posthoc_nemenyi_friedman test evaluated over all runs (sampling-Continue).

Algorithm	Sampling											
	Real_lhs						Real_random					
	Hybrid-ftol vs. BU		Hybrid-ftol vs. Without BU		Hybrid-ftol vs. Hybrid-cvtol		Hybrid-ftol vs. BU		Hybrid-ftol vs. Without BU		Hybrid-ftol vs. Hybrid-cvtol	
	p-value	h^a^	p-value	h	p-value	h	p-value	h	p-value	h	p-value	h
NSGA-II	0.59	~	0.09	~	0.82	~	0.59	~	0.09	~	0.82	~

^a^Posthoc_nemenyi_friedman: +, ~, and – presents the first method performs statistically significantly better, equal, and worse than the second approach.

**Table 38:** Summary of p value of the posthoc_nemenyi_friedmantest evaluated over all runs (crossover).

Algorithm	Crossover											
	Uniform						real_sbx					
	Hybrid-cvtol vs. BU		Hybrid-cvtol vs. Without BU		BU vs. Without BU		Hybrid-cvtol vs. BU		Hybrid-cvtol vs. Without BU		BU vs. Without BU	
	p-value	h^a^	p-value	h	p-value	h	p-value	h	p-value	h	p-value	h
NSGA-II	0.67	~	0.12	~	0.67		0.35	~	0.03	+	0.67	~

^a^Posthoc_nemenyi_friedman: +, ~, and – presents the first method performs statistically significantly better, equal, and worse than the second approach.

**Table 39:** Summary of p value of the posthoc_nemenyi_friedmantest evaluated over all runs (crossover-Continue).

Algorithm	Crossover											
	Uniform						real_sbx					
	Hybrid-ftol vs. BU		Hybrid-ftol vs. Without BU		Hybrid-ftol vs. Hybrid-cvtol		Hybrid-ftol vs. BU		Hybrid-ftol vs. Without BU		Hybrid-ftol vs. Hybrid-cvtol	
	p-value	h^a^	p-value	h	p-value	h	p-value	h	p-value	h	p-value	h
NSGA-II	0.12	~	0.005	+	0.67	~	0.35	~	0.03	+	0.90	~

^a^Posthoc_nemenyi_friedman: +, ~, and – presents the first method performs statistically significantly better, equal, and worse than the second approach.

This example provides valuable insights into the application of the BU method in addressing complex, large-scale, constrained optimization problems. The extension of the original single-objective problem to a multi-objective setting showcases the flexibility of the proposed methodology. The utilization of hypervolume as a performance indicator reaffirms the substantial improvements achieved by the hybrid approaches. Overall, this study contributes not only to the optimization of the Cantilevered Stepped Design problem but also sheds light on the broader potential of the BU method in tackling diverse engineering optimization challenges.

#### Multi-objective car-side impact problem formulation

In scenarios where the black-box constraints of an optimization problem exhibit highly complex behavior, the direct evaluation of these constraints can be prohibitively time-consuming. In such instances, the use of surrogates becomes a valuable strategy to mitigate the complexity and expedite the optimization process. Surrogates, effectively approximating the actual constraints, enable the optimization algorithm to learn from previous evaluations and make informed decisions. The surrogate model, if reducible to a single variable, provides an opportunity to apply the BU approach to solve the constrained surrogate model efficiently, as detailed in the Appendix.

The car side impact problem stands out as a prime example of a complex and time-consuming optimization challenge, focusing on the multi-objective optimization of a vehicle's side impact crashworthiness 50. Repairing variables ${X}_{2} and{ X}_{3}$ are identified for this problem. Various evolutionary algorithms, including NSGA-II, NSGA-III, UNSGA-III, and AGEMOEA, are implemented to explore the solution space. Tables 40, 41, 42, 43, 44, 45 and 46 present the results of the non-dominated solutions obtained by these methods.

**Table 40:** Results of different methods on the multi-objective car-side impact problem (HV values-sampling).

Algorithm	Sampling											
	real_random						Real_lhs					
	BU step	Best	Mean	Median	Worst	St. Dev	BU step	Best	Mean	Median	Worst	St. Dev
NSGA-II	None^a^	0.249	0.244	0.2466	0.2398	0.0038	None	0.260	0.217	0.213	0.180	0.0283
	BU	0.770	0.756	0.76	0.733	0.0132	BU	0.767	0.743	0.750	0.700	0.0228
	Hybrid-cvtol	**0.818**	0.800	0.7985	0.790	0.0094	Hybrid-cvtol	**0.814**	0.795	**0.799**	0.768	0.0151
	Hybrid-ftol	0.803	**0.803**	**0.803**	**0.802**	0.0004	Hybrid-ftol	0.806	**0.797**	0.797	**0.785**	0.0075
NSGA-III	None	0.259	0.165	0.1333	0.1225	0.0520	None	0.263	0.186	0.154	0.136	0.0505
	BU	0.818	0.803	0.805	0.778	0.0141	BU	0.824	0.809	0.813	0.785	0.0132
	Hybrid-cvtol	0.850	0.826	0.826	0.810	0.0141	Hybrid-cvtol	**0.853**	0.829	0.824	0.810	0.0162
	Hybrid-ftol	**0.858**	**0.852**	**0.852**	**0.845**	0.004	Hybrid-ftol	0.841	**0.833**	**0.837**	**0.819**	0.008
UNSGA-III	None	0.214	0.191	0.1996	0.1575	0.0195	None	0.182	0.141	0.149	0.074	0.0361
	BU	0.815	0.802	0.80	0.788	0.0090	BU	**0.833**	0.805	0.803	0.790	0.0146
	Hybrid-cvtol	0.823	0.811	0.816	0.791	0.0110	Hybrid-cvtol	0.830	**0.819**	**0.827**	**0.796**	0.0133
	Hybrid-ftol	**0.816**	**0.810**	**0.810**	**0.803**	0.004	Hybrid-ftol	0.829	0.822	0.826	0.811	0.006
AGEMOEA	None	0.272	0.226	0.222	0.1937	0.0265	None	0.276	0.224	0.251	0.163	0.0481
	BU	0.849	0.801	0.794	0.771	0.0257	BU	0.804	0.777	0.773	0.763	0.0140
	Hybrid-cvtol	**0.849**	**0.828**	0.819	0.812	0.0145	Hybrid-cvtol	**0.853**	0.833	0.833	0.816	0.0147
	Hybrid-ftol	0.835	0.827	**0.826**	**0.816**	0.006	Hybrid-ftol	0.841	**0.834**	**0.838**	**0.823**	0.006
NSDE	None	0.825	0.803	0.811	0.770	0.018	None	0.830	0.773	0.782	0.707	0.045
	BU	0.812	0.780	0.771	0.747	0.026	BU	0.777	0.757	0.767	0.734	0.018
	Hybrid-cvtol	**0.818**	**0.810**	**0.816**	**0.794**	0.008	Hybrid-cvtol	**0.827**	**0.813**	**0.811**	**0.803**	0.008
	Hybrid-ftol	0.808	0.798	0.793	0.791	0.007	Hybrid-ftol	0.823	0.809	0.809	0.791	0.011
NSDER	None	0.782	0.737	0.737	0.704	0.029	None	0.816	0.805	0.812	0.774	0.015
	BU	0.790	0.759	0.767	0.734	0.021	BU	0.794	0.782	0.781	0.776	0.006
	Hybrid-cvtol	**0.829**	**0.816**	**0.817**	**0.809**	0.006	Hybrid-cvtol	**0.840**	**0.809**	**0.805**	**0.791**	0.016
	Hybrid-ftol	0.823	0.806	0.809	0.791	0.104	Hybrid-ftol	0.808	0.798	0.793	**0.791**	0.007

“None” means no BU method or hybrid is used and only feasibility rules as an explicit constraint handling method is worked alone.

Significant values are in bold.

**Table 41:** Results of different methods on the multi-objective car side design problem (HV values-crossover).

Algorithm	Crossover											
	real_sbx						Uniform					
	BU step	Best	Mean	Median	Worst	St. Dev	BU step	Best	Mean	Median	Worst	St. Dev
NSGA-II	None	0.237	0.189	0.180	0.170	0.024	None	0.309	0.264	0.271	0.166	0.051
	BU	0.765	0.741	0.750	0.697	0.025	BU	0.773	0.756	0.761	0.732	0.013
	Hybrid-cvtol	**0.810**	0.798	0.798	0.776	0.012	Hybrid-cvtol	**0.806**	0.798	0.799	0.786	0.007
	Hybrid-ftol	0.808	**0.801**	**0.805**	**0.790**	0.006	Hybrid-ftol	0.801	**0.799**	**0.800**	**0.793**	0.003
NSGA-III	None	0.254	0.187	0.186	0.120	0.054	None	0.237	0.2064	0.212	0.164	0.025
	BU	0.815	0.806	0.811	0.793	0.008	BU	0.816	0.785	0.772	0.766	0.021
	Hybrid-cvtol	**0.853**	0.824	0.829	0.781	0.023	Hybrid-cvtol	**0.844**	0.828	0.828	0.815	0.008
	Hybrid-ftol	0.833	**0.826**	**0.830**	**0.814**	0.007	Hybrid-ftol	0.833	**0.829**	**0.831**	**0.822**	0.004
UNSGA-III	None	0.188	0.181	0.182	0.174	0.004	None	0.255	0.226	0.218	0.198	0.020
	BU	0.797	0.783	0.788	0.767	0.011	BU	0.828	0.801	0.802	0.775	0.016
	Hybrid-cvtol	**0.851**	0.834	0.829	0.816	0.013	Hybrid-cvtol	**0.834**	0.824	**0.828**	0.808	0.008
	Hybrid-ftol	0.842	**0.835**	**0.839**	**0.824**	0.006	Hybrid-ftol	0.830	**0.825**	0.825	**0.819**	0.003
AGEMOEA	None	0.267	0.225	0.217	0.193	0.030	None	0.278	0.252	0.270	0.192	0.032
	BU	0.810	0.781	0.791	0.729	0.027	BU	0.799	0.788	0.791	0.777	0.008
	Hybrid-cvtol	**0.856**	**0.839**	**0.843**	0.826	0.011	Hybrid-cvtol	**0.853**	**0.839**	**0.851**	0.817	0.016
	Hybrid-ftol	0.845	0.838	0.838	**0.830**	0.005	Hybrid-ftol	0.848	**0.839**	0.838	**0.827**	0.007
NSDE	None	0.814	0.804	0.804	0.791	0.008	None	0.810	0.805	0.805	0.799	0.003
	BU	0.796	0.782	0.781	0.763	0.012	BU	0.789	0.783	0.782	0.774	0.005
	Hybrid-cvtol	**0.815**	**0.810**	**0.810**	**0.804**	0.003	Hybrid-cvtol	**0.812**	**0.810**	**0.811**	**0.808**	0.001
	Hybrid-ftol	0.802	0.798	0.798	0.793	0.003	Hybrid-ftol	0.799	0.798	0.798	0.796	0.001
NSDER	None	0.755	0.739	0.738	0.718	0.013	None	0.747	0.740	0.739	0.730	0.0060
	BU	0.772	0.760	0.760	0.745	0.009	BU	0.765	0.760	0.760	0.754	0.0037
	Hybrid-cvtol	0.819	**0.816**	**0.816**	**0.812**	0.002	Hybrid-cvtol	0.816	0.816	0.816	**0.815**	0.0005
	Hybrid-ftol	**0.872**	0.813	0.811	0.737	0.048	Hybrid-ftol	**0.846**	**0.823**	**0.836**	0.781	0.0251

**Table 42:** Summary of the p-value of the Friedman rank test over all runs.

Algorithm	Sampling				Crossover			
	Real_lhs		Real_random		*Uniform*		*real_sbx*	
	p-value	Statistic value	p-value	Statistic value	p-value	Statistic value	p-value	Statistic value
NSGA-II	0.0067	10.0	0.0067	10.0	0.0067	10.0	0.0067	10.0
NSGA-III	0.0149	8.40	0.0149	8.40	0.0067	10.0	0.0149	8.40
UNSGA-III	0.0223	7.60	0.02237	7.60	0.0067	10.0	0.0067	10.0
AGEMOEA	0.0223	7.60	0.0149	8.40	0.0067	10.0	0.0067	10.0
NSDE	0.032	8.75	0.050	7.73	0.0018	15.0	0.002	14.03
NSDER	0.054	7.62	0.001	15.0	0.003	13.56	0.006	12.11

**Table 43:** Summary of the p-value of the posthoc_nemenyi_friedman test evaluated over all runs (sampling).

Algorithm	Sampling											
	Real_lhs						Real_random					
	Hybrid-cvtol vs. BU		Hybrid-cvtol vs. Without BU		BU vs. Without BU		Hybrid-cvtol vs. BU		Hybrid-cvtol vs. Without BU		BU vs. Without BU	
	p-value	h^a^	p-value	h	p-value	h	p-value	h	p-value	h	p-value	h
NSGA-II	0.31	~	0.01	+	0.59	~	0.51	~	0.06	~	0.67	~
NSGA-III	0.51	~	0.06	~	0.67	~	0.67	~	0.12	~	0.67	~
UNSGA-III	0.82	~	0.06	~	0.35	~	0.22	~	0.01	+	0.67	~
AGEMOEA	0.51	~	0.06	~	0.67	~	0.67	~	0.04	+	0.43	~
NSDE	0.03	+	0.35	~	0.67	~	0.03	+	0.82	~	0.22	~
NSDER	0.04	+	0.74	~	0.35	~	0.12	~	0.005	+	0.67	~

^a^Posthoc_nemenyi_friedman: +, ~, and – presents the first method performs statistically significantly better, equal, and worse than the second approach.

**Table 44:** Summary of the p-value of the posthoc_nemenyi_friedman test evaluated over all runs (sampling).

Algorithm	Sampling											
	Real_lhs						Real_random					
	Hybrid-ftol vs. BU		Hybrid-ftol vs. Without BU		Hybrid-cvtol vs Hybrid-ftol		Hybrid-ftol vs. BU		Hybridfvtol vs. Without BU		Hybrid-cvtol vs Hybrid-ftol	
	p-value	h^a^	p-value	h	p-value	h	p-value	h	p-value	h	p-value	h
NSGA-II	0.20	~	0.007	+	0.90	~	0.22	~	0.01	+	0.90	~
NSGA-III	0.22	~	0.01	+	0.90	~	0.12	~	0.005	+	0.67	~
UNSGA-III	0.82	~	0.06	~	0.90	~	0.51	~	0.90	~	0.06	~
AGEMOEA	0.22	~	0.01	+	0.90	~	0.74	~	0.06	~	0.90	~
NSDE	0.35	~	0.90	~	0.67	~	0.03	+	0.82	~	0.22	~
NSDER	0.59	~	0.90	~	0.51	~	0.67	~	0.12	~	0.67	~

^a^Posthoc_nemenyi_friedman: +, ~, and – presents the first method performs statistically significantly better, equal, and worse than the second approach.

**Table 45:** Summary of the p-value of the posthoc_nemenyi_friedman test evaluated over all runs (Crossover).

Algorithm	Crossover											
	Uniform						real_sbx					
	Hybrid-cvtol vs. BU		Hybrid-cvtol vs. Without BU		BU vs. Without BU		Hybrid-cvtol vs. BU		Hybrid-cvtol vs. Without BU		BU vs. Without BU	
	p-value	h^a^	p-value	h	p-value	h	p-value	h	p-value	h	p-value	h
NSGA-II	0.45	~	0.03	+	0.59	~	0.45	~	0.03	+	0.59	~
NSGA-III	0.51	~	0.06	~	0.67	~	0.82	~	0.12	~	0.51	~
UNSGA-III	0.67	~	0.12	~	0.67	~	0.82	~	0.06	~	0.35	~
AGEMOEA	0.28	~	0.02	+	0.67	~	0.22	~	0.01	+	0.67	~
NSDE	0.005	+	0.67	~	0.12	~	0.005		0.51	~	0.22	~
NSDER	0.51	~	0.06	~	0.67	~	0.35	~	0.013		0.51	~

^a^Posthoc_nemenyi_friedman: +, ~, and – presents the first method performs statistically significantly better, equal, and worse than the second approach.

**Table 46:** Summary of the p-value of the posthoc_nemenyi_friedman test evaluated over all runs (Crossover).

Algorithm	Crossover											
	Uniform						real_sbx					
	Hybrid-ftol vs. BU		Hybrid-ftol vs. Without BU		Hybrid-cvtol vs Hybrid-ftol		Hybrid-ftol vs. BU		Hybridfvtol vs. Without BU		Hybrid-cvtol vs Hybrid-ftol	
	p-value	h^a^	p-value	h	p-value	h	p-value	h	p-value	h	p-value	h
NSGA-II	0.12	~	0.003	+	0.87	~	0.12	~	0.003	+	0.87	~
NSGA-III	0.22	~	0.01	+	0.90	~	0.35	~	0.01	+	0.82	~
UNSGA-III	0.12	~	0.005	+	0.67	~	0.82	~	0.06	~	0.90	~
AGEMOEA	0.43	~	0.04	+	0.90	~	0.51	~	0.06	~	0.90	~
NSDE	0.67	~	0.67	~	0.12	~	0.51	~	0.90	~	0.22	~
NSDER	0.22	~	0.013	+	0.90	~	0.82	~	0.12	~	0.82	~

^a^Posthoc_nemenyi_friedman: +, ~, and – presents the first method performs statistically significantly better, equal, and worse than the second approach.

The results underscore the superiority of the hybrid methods, implemented with the proposed evolutionary algorithms, in terms of diversity. The hybrid approaches exhibit a well-distributed population across the entire search space, leading to enhanced performance. While the application of the BU approach alone may face efficiency challenges, particularly in the context of a three-objective optimization problem, the hybrid approach proves adept at overcoming these difficulties. Statistical analyses of the results reveal that, in some cases and with different algorithms, the BU method may not be as efficient, yet the hybrid approach consistently outperforms it, signifying the added benefits of the hybridization strategy.

Comparative statistical tests emphasize the significant advantage of the hybrid approaches over the BU method. Notably, although some statistical tests indicate no significant difference between the hybrid and BU methods, the hybrid method consistently achieves higher hypervolume (HV) values. This suggests that, even when statistical significance is not apparent, the hybrid approach provides a qualitative improvement in solution quality.

In conclusion, the exploration of the car side impact problem demonstrates the efficacy of hybrid approaches in addressing complex, multi-objective optimization challenges with intricate black-box constraints. The incorporation of surrogates and the application of the BU method within a hybrid framework contribute to improved diversity and solution quality. These findings not only advance the optimization of the car side impact problem but also offer valuable insights into the broader applicability of hybrid evolutionary algorithms in addressing real-world, complex optimization scenarios.

## Discussion

The BU method is an implicit constraint-handling technique coupled with an explicit constraint-handling technique that aims to cut the infeasible search space over iterations to find the feasible region faster. Since optimization is an iterative process whose boundaries can be changed during the process, the *i*-th decision variable boundaries in each iteration exhibit a dynamic nature. By applying this philosophy to boost the optimization process in the BU method, the bounds of selected variable (s) are repaired (repairing variables) to satisfy the constraints. As a result, new solutions are created within the updated boundary of the latest search space. The above-mentioned process helps the algorithm search for a solution within the feasible region instead of the whole area. Although the BU method directs the search operator to the feasible space and reaches the feasible region in a reasonable time, it twists the search space, making the optimization problem more challenging. As such, the results are not always accurate, and the algorithm may result in premature convergence. Therefore, in this paper, two “hybrid” switching mechanisms are proposed. In the proposed approach, the BU method is applied to the problem after initializing the algorithm, and in this phase, a threshold tolerance is set. The BU method optimizes the problem based on the threshold tolerances so that the population has zero constraint violations or there is no more change in the objective space. Then, the optimization problem is continued with the BU method.

The results suggest that:

- Advantageously, the hybrid method can be used with and without the BU method. In the first phase of the hybrid method, the BU method helps the optimization algorithm to avoid searching inside the infeasible region and boosts it to explore the feasible area more efficiently. This way, the optimization algorithm finds the feasible area with fewer FEs. However, because the BU brings about changes to the landscape of the search space, it may result in premature convergence. Hence, in the second phase of the hybrid method, the optimization algorithm searches the feasible area without using the BU approach.
- GA has the least standard deviation among the other algorithms for the single-objective function optimization problem.
- The statistical tests for single-objective optimization problems show there is a significant difference between hybrid methods and two other methods (with BU and without BU) using GA. Moreover, GA works well for this method and boosts optimization problems. Due to the fact that GA can benefit from different operator settings, such as crossover and sampling, better results by choosing the appropriate operator setting may be achieved.
- Regarding the multi-objective optimization problems, the hybrid method could find more non-dominated solutions with better distribution compared to with and without the BU methods. The BNH test problems indicated that the hybrid method produced promising results for IGD and HV values compared to with BU and without BU approaches.
- Although the BU and hybrid methods outperformed the other algorithms in single- and multi-objective optimization, they are still case-dependent, and there are EAs that are not suitable in some cases.
- By boosting the optimization process, the hybrid method finds the first and whole populations much faster than the classic BU and without BU methods for most problems (i.e., the single- or multi-objective optimization problems of this paper). Moreover, regarding the car side impact design problem, the hybrid method obtained good diversity, whereas without the BU method searched for solutions in a specific feasible space.
- For some case studies, although the hybrid method resulted in the same (or close-to-final) solution as the two other methods, it requires fewer FEs to converge to a solution.
- Compared to unconstrained optimization problems, constrained optimization problems are more challenging since many infeasible regions appear in the search space (meaning the hit ratio is low). This makes solving a constrained problem more challenging, especially highly constrained problems that can lead to difficulties such as convergence-, diversity-, and feasibility-hardness. Therefore, finding just one feasible solution is a significant achievement. The primary goal of the BU method is to minimize the search space by narrowing down the variable range and eliminating infeasible options. To compare the effectiveness of the proposed approach, the first feasible solution found by different methods is compared to one another, and the whole population is tracked against generations. The hybrid method uses this philosophy to switch the optimization process and avoid reaching premature convergence. On the other hand, once the whole feasible area is found by the BU method, the final solution (global) could be achieved with fewer FEs in a reasonable time.
- Regarding the multi-objective car side impact design problem, the hybrid approach works significantly better than without the BU method when incorporated in the employed multi-objective algorithms. It is worth mentioning that although there is no significant difference in the final HV values between the hybrid and BU methods, as mentioned earlier, the hybrid method could achieve the final solution in fewer FEs.
- In scenarios where explicit formulation of constraints is challenging, the use of surrogate models, also known as metamodels, proves to be a valuable solution. Surrogate models have demonstrated effectiveness in situations where deriving explicit formulations for constraints is impractical. These models serve as approximations of intricate simulations, providing an efficient means to evaluate constraints during optimization without the computational overhead of running resource-intensive simulations. For instance, consider optimization problems associated with finite element models, such as those involving nodal displacements and bar stresses in truss optimization. In these cases, surrogate models can be strategically trained to emulate the intricate behavior observed in simulation results. This approach becomes particularly advantageous when the direct formulation of these constraints proves to be impractical due to their complexity or reliance on computationally expensive procedures.

In EAs, sampling and crossover are critical factors that can strongly affect convergence speed and are often used in noisy optimization problems where reducing the negative impact of noise is crucial. Moreover, sampling is a popular strategy for dealing with noise. Therefore, experiments with different sampling operators, including real random sampling, Latin hypercube sampling, and two different real-SBX and uniform crossovers, were conducted to reduce the noise impact.

## Limitations

Although this study provides valuable insights into optimization problems, there are certain limitations to this work. One significant limitation of this work lies in the relatively small size of variables in the problems. It should be noted that this study aims to contribute by addressing a specific aspect of the optimization problem. In this study, the benchmark libraries have not been used and some specific problems with solvable constraints with respect to at least one variable have been used. While the limited number of variables in our optimization problems may restrict the immediate applicability of our findings to large-scale size problems, it is clear the insights gained from this research serve as a valuable foundation for further research in the field.

Another limitation of this study is related to the experimental section, where the algorithms compared with the proposed algorithm are not the most recent. It is important to note that the aim of this work is not focused on proposing and developing state-of-the-art algorithms; rather, the advancement of constraint handling techniques and the development of the BU approach are being concentrated on. Consequently, well-known algorithms such as NSGA-II in the analysis have been included. However, as a future study, it would be valuable to consider incorporating state-of-the-art algorithms to provide a more comprehensive evaluation of our proposed approach in comparison to the latest advancements in the field.

Moreover, while the current selection of repairing variables is designed to be applicable even in scenarios with multiple disjoint feasible regions, the evaluation of constraints can pose challenges in situations where the behavior is non-trivial. In such cases, alternative approaches to repairing variable selection, involving book-keeping or linking of these disjoint feasible ranges, may be necessary. This intriguing aspect has been highlighted and it is proposed as a subject for future investigation, intending to delve deeper into the nuanced evaluation of constraints in conditions assessment.

## Conclusion

This study presents an extension of the work proposed by^6^ for solving constrained optimization problems efficiently. The proposed methods use an implicit constraint handling approach called boundary updating (BU), which focuses on directly handling constraints and is coupled with other explicit constraint-handling techniques (here, feasibility rules).

In this study, two augmented versions of the BU method were applied to single-objective, multi-objective, and many-objective constrained optimization problems, including benchmarks and real-world problems. To accomplish this goal, two thresholds are considered, each representing different switching method. In the initial method, the optimization process shifts to a state that does not involve the BU approach once constraint violations reach zero. In the second approach, the optimization process enters a phase without BU methods when there is no longer any observed change in the objective space. For single-objective constrained optimization problems, popular algorithms like GA, DE, ES, SRES, and BRKGA were employed, and NSGA-II, a renowned algorithm, was used for bi-objective problems. For the multi-objective constrained optimization problems, NSGA-III, UNSGA-III, and AGEMOEA were applied. The results show that the hybrid approach performs significantly better than with- and without the BU methods, and can find better solutions with fewer iterations.

As a future study, it is suggested that the proposed BU method be coupled with other constraint-handling techniques, for example different types of penalty methods with different objective functions. Also, additional test problems in the literature, such as many-objective and dynamic functions, could be considered for further validating the proposed hybrid approach. After BU generalization, it can also be applied to benchmark libraries such as CEC, NEVERGRAD, BBOB, GNBG, and DIRECTGOLib. Although BU is not defined for any specific optimization algorithms, implementing BU to a wider range of optimization algorithms can be investigated in another future study. Moreover, as a prospective avenue for future research, exploring the applicability of the proposed approach to constrained problems featuring multiple disconnected regions is a promising direction. Many constrained optimization scenarios exhibit this characteristic, with only certain regions potentially close to the true optimum. Investigating the effectiveness and adaptability of our approach in addressing such complex problem structures holds significant potential for enhancing the robustness and versatility of the proposed methodology. This important consideration will be a key focus in our forthcoming studies.

## Data Availability

All data used in this study are simulated and not publicly available. The datasets were generated solely for the purpose of conducting the research outlined in this manuscript. Simulated data files are not deposited in any public repository or third-party platform. However, upon reasonable request, and subject to any applicable restrictions, the corresponding author can provide the simulated data used in this study. Also, our code has been executed using the open-source Python library, Pymoo (https://pymoo.org/index.html) and Pymoode (https://pymoode.readthedocs.io/en/latest/).

## Appendix

G01 formulation:

$${f}_{1}\left(x\right)=5\times \left({x}_{1}+{x}_{2}+{x}_{3}+{x}_{4}\right)-5\times \left({x}_{1}^{2}+{x}_{2}^{2}+{x}_{3}^{2}+{x}_{4}^{2}\right)$$

39 $$-({x}_{5}+{x}_{6}+{x}_{7}+{x}_{8}+{x}_{9}+{x}_{10}+{x}_{11}+{x}_{12}+{x}_{13})$$

S.t.

40 $${g}_{1}\left(x\right)=2\times {x}_{1}+2\times {x}_{2}+{x}_{10}+{x}_{11}\le 10$$

41 $${g}_{2}\left(x\right)=2\times {x}_{1}+2\times {x}_{3}+{x}_{10}+{x}_{12}\le 10$$

42 $${g}_{3}\left(x\right)=2\times {x}_{2}+2\times {x}_{3}+{x}_{11}+{x}_{12}\le 10$$

43 $${g}_{4}\left(x\right)=-8\times {x}_{1}+{x}_{10}\le 0$$

44 $${g}_{5}\left(x\right)=-8\times {x}_{2}+{x}_{11}\le 0$$

45 $${g}_{6}\left(x\right)=-8\times {x}_{3}+{x}_{12}\le 0$$

46 $${g}_{7}\left(x\right)=-2\times {x}_{4}-{x}_{5}+{x}_{10}\le 0$$

47 $${g}_{8}\left(x\right)=-2\times {x}_{6}-{x}_{7}+{x}_{11}\le 0$$

48 $${g}_{9}\left(x\right)=-2\times {x}_{8}-{x}_{9}+{x}_{12}\le 0$$

49 $$0\le {x}_{1}\cdots {x}_{9}\le 1, 0\le {x}_{10},{x}_{11},{x}_{12}\le 100, 0\le {x}_{13}\le 1$$

Car-side problem formulation:

50 $${f}_{1}\left(x\right)=1.98+4.90\times {x}_{1}+6.67\times {x}_{2}+6.98\times {x}_{3}+4.01\times {x}_{4}+1.78\times {x}_{5}+2.73\times {x}_{7}$$

51 $${g}_{1}\left(x\right)=1.16-0.3717\times {x}_{2}\times {x}_{4}-0.00931{\times x}_{2}\times {x}_{10}-0.484\times {x}_{3}\times {x}_{9}+0.01343\times {x}_{6}\times {x}_{10}$$

52 $${g}_{2}\left(x\right)=46.36-9.9\times {x}_{2}-12.9\times {x}_{1}\times {x}_{8}+0.1107\times {x}_{3}\times {x}_{10}$$

53 $${g}_{3}\left(x\right)=33.86+2.95\times {x}_{3}+0.1792\times {x}_{10}-5.057\times {x}_{1}\times {x}_{2}-11.0\times {x}_{2}\times {x}_{8}-0.021\times {x}_{5}\times {x}_{10}-9.98\times {x}_{7}\times {x}_{8}+22.0\times {x}_{8}\times {x}_{9}$$

54 $${g}_{4}\left(x\right)=28.98+3.818\times {x}_{3}-4.2\times {x}_{1}\times {x}_{2}-5.0207\times {x}_{5}\times {x}_{10}+6.63\times {x}_{6}\times {x}_{9}-7.7\times {x}_{7}\times {x}_{8}+0.32\times {x}_{9}\times {x}_{10}$$

55 $${g}_{5}\left(x\right)=0.261-0.0159\times {x}_{1}\times {x}_{2}-0.188\times {x}_{1}\times {x}_{8}-0.019\times {x}_{2}\times {x}_{7}+0.0144\times {x}_{3}\times {x}_{5}+0.0008757\times {x}_{5}\times {x}_{10}+0.08045\times {x}_{6}\times {x}_{9}+0.00139\times {x}_{8}\times {x}_{11}+0.00001575\times {x}_{10}\times {x}_{11}$$

56 $${g}_{6}\left(x\right)=0.214+0.00817\times {x}_{5}-0.0131\times {x}_{1}\times {x}_{8}-0.0704\times {x}_{1}\times {x}_{9}+0.03099\times {x}_{2}\times {x}_{6}-0.018\times {x}_{2}\times {x}_{7}+0.0208\times {x}_{3}\times {x}_{8}+0.121\times {x}_{3}\times {x}_{9}-0.00364\times {x}_{5}\times {x}_{6}+0.0007715\times {x}_{5}\times {x}_{10}-0.0005354\times {x}_{6}\times {x}_{10}+0.00121\times {x}_{8}\times {x}_{11}+0.00184\times {x}_{9}\times {x}_{10}-0.02\times {x}_{2}^{2}$$

57 $${g}_{7}\left(x\right)=0.74-0.61\times {x}_{2}-0.163\times {x}_{3}\times {x}_{8}+0.001232\times {x}_{3}\times {x}_{10}-0.166\times {x}_{7}\times {x}_{9}+0.227\times {x}_{2}^{2}$$

58 $${g}_{8}\left(x\right)=4.72-0.5\times {x}_{4}-0.19\times {x}_{2}\times {x}_{3}-0.0122\times {x}_{4}\times {x}_{10}+0.009325\times {x}_{6}\times {x}_{10}+0.000191\times {x}_{11}^{2}$$

59 $${g}_{9}\left(x\right)=10.58-0.674\times {x}_{1}\times {x}_{2}-1.95\times {x}_{2}\times {x}_{8}+0.02054\times {x}_{3}\times {x}_{10}-0.0198\times {x}_{4}\times {x}_{10}+0.028\times {x}_{6}\times {x}_{10}$$

60 $${g}_{10}=16.45-0.489\times {x}_{3}\times {x}_{7}-0.843\times {x}_{5}\times {x}_{6}+0.0432\times {x}_{9}\times {x}_{10}-0.0556\times {x}_{9}\times {x}_{11}-0.000786\times {x}_{11}^{2}$$

61 $$0.5\le {x}_{1},{x}_{3},{x}_{4}\le 1.5, 0.45\le {x}_{2}\le 1.35, 0.875\le {x}_{5}\le 2.625, 0.4\le {x}_{6},{x}_{7}\le 1.2, 0.19\le {x}_{8},{x}_{9}\le 0.35, -30\le {x}_{10},{x}_{11}\le 30$$

Speed reducer formulation:

62 $$\mathrm{min }z=0.785\times {x}_{1}\times {x}_{2}^{2}\times (3.33\times {x}_{3}^{2}+14.9334\times {x}_{3}-43.0934)-1.508\times {x}_{1}\times ({x}_{6}^{2}+{x}_{7}^{2})+7.4777\times ({x}_{6}^{3}+{x}_{7}^{3})+ 0.7854\times ({x}_{4}\times {x}_{6}^{2}+{x}_{5}\times {x}_{7}^{2})$$

63 $${\text{S}}.{\text{t}}.$$

64 $$27/({x}_{1}\times {x}_{2}^{2}\times {x}_{3})\le 1$$

65 $$397.5/{x}_{1}\times {x}_{2}^{2}\times {x}_{3}^{2}\le 1$$

66 $$1.93\times {x}_{4}^{3}/{x}_{2}\times {x}_{3}{\times x}_{6}^{4}\le 1$$

67 $$1.93\times {x}_{5}^{3}/{(x}_{2}\times {x}_{3}\times {x}_{7}^{4})\le 1$$

68 $$\sqrt{{\left(745\times \frac{{x}_{4}}{{x}_{2}\times {x}_{3}}\right)}^{2}+1.69\times {10}^{7}}/(110\times {x}_{6}^{3})\le 1$$

69 $$\sqrt{{\left(745\times \frac{{x}_{5}}{{x}_{2}\times {x}_{3}}\right)}^{2}+1.575\times {10}^{8}}/(85\times {x}_{7}^{3})\le 1$$

70 $$({x}_{2}\times {x}_{3})/40\le 1$$

71 $$(5\times {x}_{2})/{x}_{1}\le 1$$

72 $${x}_{1}/(12\times {x}_{2})\le 1$$

73 $$(1.5\times {x}_{6}+1.9)/{x}_{4}\le 1$$

74 $$(1.1\times {x}_{7}+1.9)/{x}_{5}\le 1$$

$$2.6\le {x}_{1}\le \mathrm{3.6,0.7}\le {x}_{2}\le 0.8, 17\le {x}_{3}\le 28,$$

$$7.3\le {x}_{4}\le 8.3, 7.8\le {x}_{5}\le 8.3, 2.9\le {x}_{6}\le 3.9,$$

75 $$5.0\le {x}_{7}\le 5.5$$

OSY formulation:

76 $$\mathrm{min }{f}_{1}\left(x\right)=-[25\times {\left({x}_{1}-2\right)}^{2}+{\left({x}_{2}-2\right)}^{2}+{\left({x}_{3}-1\right)}^{2}+{\left({x}_{4}-4\right)}^{2}+{\left({x}_{5}-1\right)}^{2}]$$

$$\mathrm{min }{f}_{2}\left(x\right)={x}_{1}^{2}+{x}_{2}^{2}+{x}_{3}^{2}+{x}_{4}^{2}+{x}_{5}^{2}+{x}_{6}^{2}$$

77 $${\text{S}}.{\text{t}}.$$

78 $${C}_{1}\left(x\right)={x}_{1}+{x}_{2}-2\ge 0$$

79 $${C}_{2}\left(x\right)={6-x}_{1}-{x}_{2}\ge 0$$

80 $${C}_{3}\left(x\right)={2-{x}_{2}+x}_{1}\ge 0$$

81 $${C}_{4}\left(x\right)={2-{x}_{1}+3\times x}_{2}\ge 0$$

82 $${C}_{5}\left(x\right)={4-{{(x}_{3}-3)}^{2}-x}_{4}\ge 0$$

83 $${C}_{6}\left(x\right)={{{(x}_{5}-3)}^{2}+x}_{6}-4\ge 0$$

84 $$0\le {x}_{1},{x}_{2},{x}_{6}\le 10\cdots 1\le {x}_{3},{x}_{5}\le 5\cdots 1\le {x}_{4}\le 6$$

BNH formulation:

85 $$\mathrm{min }{f}_{1}\left(x\right)=4\times {x}_{1}^{2}+4\times {x}_{2}^{2}$$

86 $$\mathrm{min }{f}_{2}\left(x\right)={({x}_{1}-5)}^{2}+{({x}_{2}-5)}^{2}$$

87 $${\text{S}}.{\text{t}}.$$

88 $${C}_{1}\left(x\right)={({x}_{1}-5)}^{2}+{x}_{2}^{2}\le 25$$

89 $${C}_{2}\left(x\right)={({x}_{1}-8)}^{2}+{({x}_{2}+3)}^{2}\ge 7.7$$

90 $$0\le {x}_{1}\le 5\cdots 0\le {x}_{2}\le 3$$

Cantilevered beam design problem:

91 $$\mathrm{Min }{{\text{f}}}_{1}\left({\text{x}}\right)={100\times ({\text{x}}}_{5}\times {{\text{x}}}_{6}+{{\text{x}}}_{7}\times {{\text{x}}}_{8}+{{\text{x}}}_{9}\times {{\text{x}}}_{10}+{{\text{x}}}_{1}\times {{\text{x}}}_{2}+{{\text{x}}}_{3}\times {{\text{x}}}_{4})$$

92 $$\mathrm{Min }{{\text{f}}}_{2}\left({\text{x}}\right)=10000\times (\frac{244}{{{\text{x}}}_{5}\times {{\text{x}}}_{6}^{3}}+\frac{148}{{{\text{x}}}_{7}\times {{\text{x}}}_{8}^{3}}+\frac{76}{{{\text{x}}}_{9}\times {{\text{x}}}_{10}^{3}}+\frac{28}{{{\text{x}}}_{1}\times {{\text{x}}}_{2}^{3}}+\frac{4}{{{\text{x}}}_{3}\times {{\text{x}}}_{4}^{3}})$$

Subject to

93 $${{\text{g}}}_{1}\left({\text{x}}\right)=10.7143-\frac{{{\text{x}}}_{5}\times {{\text{x}}}_{6}^{2}}{1000}\le 0$$

94 $${{\text{g}}}_{2}\left({\text{x}}\right)=8.5714-\frac{{{\text{x}}}_{7}\times {{\text{x}}}_{8}^{2}}{1000}\le 0$$

95 $${{\text{g}}}_{3}\left({\text{x}}\right)=6.4286-\frac{{{\text{x}}}_{9}\times {{\text{x}}}_{10}^{2}}{1000}\le 0$$

96 $${{\text{g}}}_{4}\left({\text{x}}\right)=4.2857-\frac{{{\text{x}}}_{1}\times {{\text{x}}}_{2}^{2}}{1000}\le 0$$

97 $${{\text{g}}}_{5}\left({\text{x}}\right)=2.1429-\frac{{{\text{x}}}_{3}\times {{\text{x}}}_{4}^{2}}{1000}\le 0$$

98 $${{\text{g}}}_{6}\left({\text{x}}\right)={{\text{x}}}_{6}-20\times {{\text{x}}}_{5}\le 0$$

99 $${{\text{g}}}_{7}\left({\text{x}}\right)={{\text{x}}}_{8}-20\times {{\text{x}}}_{7}\le 0$$

100 $${{\text{g}}}_{8}\left({\text{x}}\right)={{\text{x}}}_{10}-20\times {{\text{x}}}_{9}\le 0$$

101 $${{\text{g}}}_{9}\left({\text{x}}\right)={{\text{x}}}_{2}-20\times {{\text{x}}}_{1}\le 0$$

102 $${{\text{g}}}_{10}\left({\text{x}}\right)={{\text{x}}}_{4}-20\times {{\text{x}}}_{3}\le 0$$

103 $${{\text{x}}}_{{\text{l}}}= [1, 30, 2.4,\mathrm{ 45,2.4,45,1},\mathrm{30,1},30]$$

104 $${{\text{x}}}_{{\text{u}}}=[5, 65, 3.1, 60, 3.1, 60, 5, 65, 5, 65]$$

Multi-objective car side formulation:

105 $${f}_{1}\left(x\right)=1.98+4.9\times {x}_{1}+6.67\times {x}_{2}+6.98\times {x}_{3}+4.01\times {x}_{4}+1.78\times {x}_{5}+0.00001\times {x}_{6}+2.73{\times x}_{7}$$

106 $${f}_{2}\left(x\right)=4.72-0.5\times {x}_{4}-0.19\times {x}_{2}\times {x}_{3}$$

107 $${f}_{3}\left(x\right)=0.5\times (10.58-0.674\times {x}_{1}\times {x}_{2}-0.67275\times {x}_{2}+16.45-0.489\times {x}_{3}\times {x}_{7}-0.843\times {x}_{5}\times {x}_{6}$$

108 $${g}_{1}\left(x\right)=1.16-0.3717\times {x}_{2}\times {x}_{4}-0.0092928\times {x}_{3}\le 1$$

109 $${g}_{2}\left(x\right)=0.261-0.0159\times {x}_{1}\times {x}_{2}-0.06486\times {x}_{1}-0.019\times {x}_{2}\times {x}_{7}+0.0144\times {x}_{3}\times {x}_{5}+0.0154464\times {x}_{6}\le 0.32$$

110 $${g}_{3}\left(x\right)=0.214+0.00817\times {x}_{5}-0.045195\times {x}_{1}-0.0135168\times {x}_{1}+0.03099\times {x}_{2}\times {x}_{6}-0.018\times {x}_{2}\times {x}_{7}+0.007176\times {x}_{3}+0.023232\times {x}_{3}-0.00364\times {x}_{5}\times {x}_{6}-0.018\times {x}_{2}^{2}\le 0.32$$

111 $${g}_{4}\left(x\right)=0.74-0.61\times {x}_{2}-0.31296\times {x}_{3}-0.031872\times {x}_{7}+0.227\times {x}_{2}^{2}\le 0.32$$

112 $${g}_{5}\left(x\right)=28.98+3.818\times {x}_{3}-4.2\times {x}_{1}\times {x}_{2}+1.2729\times {x}_{6}-2.68065\times {x}_{7}\le 32$$

113 $${g}_{6}\left(x\right)=33.86+2.95\times {x}_{3}-5.057\times {x}_{1}\times {x}_{2}-3.795\times {x}_{2}-3.4431\times {x}_{7}+1.45728\le 32$$

114 $${g}_{7}\left(x\right)=46.36-9.9\times {x}_{2}-4.4505\times {x}_{1}\le 32$$

115 $${g}_{8}\left(x\right)=4.72-0.5\times {x}_{4}-0.19\times {x}_{2}\times {x}_{3}\le 4$$

116 $${g}_{9}\left(x\right)=10.58-0.674\times {x}_{1}\times {x}_{2}-0.672\times {x}_{2}\le 9.9$$

117 $${g}_{10}\left(x\right)=16.45-0.489\times {x}_{3}\times {x}_{7}-0.843\times {x}_{5}\times {x}_{6}\le 15.7$$

118 $$0.5\le {x}_{1},{x}_{3},{x}_{4}\le 1.5, 0.45\le {x}_{2}\le 1.35, 0.875\le {x}_{5}\le 2.625, 0.4\le {x}_{6}, {x}_{7}\le 1.2,$$
